# First-Line β-Blocker Use for Hypertension in the Veterans Health Administration

**DOI:** 10.1001/jamanetworkopen.2025.29026

**Published:** 2025-08-27

**Authors:** Catherine G. Derington, Ransmond O. Berchie, April F. Mohanty, Joshua A. Jacobs, Yizhe Xu, Jordan B. King, Leah Rethy, William C. Cushman, Susan L. Zickmund, P. Michael Ho, Sridharan Raghavan, Jordana B. Cohen, Adam P. Bress

**Affiliations:** 1 Intermountain Healthcare Department of Population Health Sciences, Divisions of Health System Innovation and Research and Biostatistics, Spencer Fox-Eccles School of Medicine, University of Utah, Salt Lake City; 2 George E. Wahlen Department of Veterans Affairs Medical Center, Salt Lake City, Utah; 3 Department of Internal Medicine, Spencer Fox-Eccles School of Medicine, Division of Epidemiology, University of Utah, Salt Lake City; 4 Institute for Health Research, Kaiser Permanente Colorado, Aurora; 5 Division of Cardiology, Hospital at the University of Pennsylvania, Philadelphia; 6 Department of Preventive Medicine, University of Tennessee Health Science Center, Memphis; 7 Division of Cardiology, Department of Medicine, University of Colorado Anschutz Medical Campus, Aurora; 8 Colorado Permanente Medical Group, Denver; 9 Division of General Internal Medicine, Department of Medicine, University of Colorado Anschutz Medical Campus, Aurora; 10 Veterans Affairs Eastern Colorado Health System, Aurora; 11 Department of Biostatistics, Epidemiology, and Informatics, Perelman School of Medicine, University of Pennsylvania, Philadelphia; 12 Department of Medicine, Renal-Electrolyte and Hypertension Division, Perelman School of Medicine at the University of Pennsylvania, Philadelphia

## Abstract

**Importance:**

Starting in 2014, US guidelines have not recommended β-blockers for first-line treatment of hypertension in the absence of compelling indications due to their tolerability profile and inferior protection against stroke and mortality compared with other first-line agents. The prevalence and factors associated with this guideline-discordant practice are unknown.

**Objective:**

To estimate the prevalence of and factors associated with first-line β-blocker use among those without compelling indications for a β-blocker.

**Design, Setting, and Participants:**

Serial cross-sectional study of new users of antihypertensive medications using national, patient-level data in the Veterans Health Administration between January 1, 2000, and December 31, 2022. Patients were veterans initiating antihypertensive medications for newly diagnosed hypertension in the outpatient setting based on diagnosis codes and prescription dispenses, excluding veterans without a primary care visit in the prior year.

**Exposure:**

Initiation of antihypertensive medications for newly diagnosed hypertension.

**Main Outcomes and Measures:**

Among those without compelling indications (ie, aortic aneurysm and/or disease, angina, atrial fibrillation or arrhythmia, chronic liver disease or cirrhosis, heart failure with reduced ejection fraction, myocardial infarction, or coronary revascularization), multivariable Poisson regression was used to estimate factors associated with β-blocker initiation.

**Results:**

Of 3 138 304 included veterans (mean [SD] age 61.0 [13.0] years, 2 958 488 [94.3%] male, 149 807 [4.8%] Hispanic, 490 636 [15.6%] non-Hispanic Black, 2 028 127 [64.6%] non-Hispanic White, and and 66 798 [2.1%] other races and ethnicities ), 774 821 (24.7%) initiated a β-blocker. Overall, 684 045 patients (88.2%) who initiated a β-blocker did not have compelling indications, which decreased over time from 245 703 (91.8%) in 2000 through 2005 to 93 088 (81.5%) in 2018 to 2022. Metoprolol and carvedilol were the most commonly initiated β-blockers and increased over time, and atenolol initiation decreased over time. Among other factors, a greater prevalence ratio for β-blocker initiation was observed among older (PR, 1.05; 95% CI, 1.04-1.05), female (PR, 1.11; 95% CI, 1.09-1.14), non-Hispanic White (as reference group; Non-Hispanic Black PR, 0.74; 95% CI, 0.73-0.76; Hispanic PR, 0.75; 95% CI, 0.73-0.77; all others PR, 0.89; 95% CI, 0.86-0.92) or frail veterans (PR, 1.28; 95% CI, 1.25-1.31).

**Conclusions and Relevance:**

In this cross-sectional study, most veterans using β-blockers as a first-line treatment for hypertension did not have compelling indications, and several factors were associated with likelihood of β-blocker initiation in this group. Interventions are needed to improve initial guideline-concordant treatment for veterans with incident hypertension.

## Key Points

**Question:**

What is the prevalence of and what are the factors associated with first-line β-blocker use for hypertension among those without compelling indications for a β-blocker?

**Findings:**

In this cross-sectional study of 774 821 veterans initiating a β-blocker for hypertension between 2000 and 2022, the proportion without a compelling indication was high (88% overall) but decreased over time (92% to 82%). Among those without compelling indications, β-blocker initiation varied by sociodemographic and clinical factors, including age, sex, race and ethnicity, and history of comorbid conditions.

**Meaning:**

These findings suggest that despite national guidelines discouraging first-line β blocker use among those without compelling indications since 2014, this practice is still common, and interventions are needed to improve guideline-concordant initial treatment for hypertension.

## Teaser

This cross-sectional study evaluates the prevalence of and factors associated with first-line β-blocker use for hypertension among veterans without compelling indications for a β-blocker.

## Introduction

Guideline recommendations regarding β-blocker use for first-line treatment of high blood pressure (BP) have changed significantly over the past 3 decades. In the 1980s and 1990s, several placebo-controlled trials demonstrated their efficacy to prevent adverse cardiovascular disease (CVD) events, leading to first-line endorsement alongside thiazide and thiazide-like diuretics (hereafter, thiazides) in the Sixth and Seventh Reports of the Joint National Committee on Prevention, Detection, Evaluation, and Treatment of High Blood Pressure (JNC6 and JNC7, respectively).^1,2,3,4,5^ This changed in 2014 with the Eighth JNC Report (JNC8) recommending β-blockers as first-line only for patients with “compelling indications” supported by strong evidence of benefit.^6^ In the absence of such compelling indications, β-blockers were relegated to second-line therapy after angiotensin-converting enzyme inhibitors, angiotensin-2 receptor blockers (ARB), thiazides, and calcium channel blockers (CCB). This shift was based on trials and meta-analyses suggesting inferior stroke and mortality as well as higher discontinuation rates from adverse effects.^7,8,9,10,11,12,13^ Similar recommendations appeared in the 2017 American College of Cardiology/American Heart Association (ACC/AHA) BP guidelines and were reinforced in the 2024 AHA/American Stroke Association Guideline for the Primary Prevention of Stroke.^14,15^ In contrast, the 2018 and 2023 updates of the European Society of Hypertension (ESH) guidelines supported first-line β-blocker use for uncomplicated hypertension, citing their efficacy compared with placebo in lowering BP and preventing CVD.^16,17,18^

The quality of hypertension care for US veterans has been a major focus of the Veterans Health Administration (VA), the largest integrated health care system in the US. The Department of Veterans Affairs/Department of Defense Hypertension Clinical Practice Guidelines in 2004, 2014, and 2020 conformed with the recommendations outlined in the JNC7, JNC8, and 2017 ACC/AHA guidelines.^19,20,21^ However, in our recent analyses,^22^ we identified that β-blockers are prescribed as first-line hypertension treatment in approximately 20% of veterans who do not have a compelling indication, although this trend decreased over the 20-year study. To improve evidence-based prescribing of β-blockers for treatment of high BP, we sought to investigate the clinical factors associated with β-blocker use in a cohort of veterans initiating treatment for high BP over 23 years.

## Methods

### Data and Study Oversight

This serial cross-sectional study was conducted using national VA data containing information from clinical encounters, pharmacy dispenses, and administrative claims for all VA medical centers and outpatient clinics in 50 US states, the District of Columbia, and unincorporated US territories (data sources in the eMethods in Supplement 1). The Salt Lake City Veterans Affairs Health Care System research and development office approved this study, and the University of Utah institutional review board approved this study with a waiver of informed consent because data were deidentified. This study followed the Strengthening the Reporting of Observational Studies in Epidemiology (STROBE) reporting guidelines for cross-sectional studies.^23^

### Study Design and Population

We identified 7 195 080 veterans who initiated an antihypertensive medication in the outpatient setting between January 1, 2000, through December 31, 2022 (index identification period), and were eligible for inclusion in the current analysis. The first pharmacy fill of an oral antihypertensive medication in the index identification period defined each veteran’s index date. We further excluded veterans who filled an antihypertensive medication before the study period to remove prevalent users (1 227 150 patients). Additional exclusion criteria included (1) less than 1 outpatient hypertension diagnosis code in the 1 year before or 180 days after the index date (1 243 181 patients); (2) being aged less than 18 years (35 patients); (3) data inconsistencies (eg, missing date of birth) (24 122 patients); and (4) less than 1 primary care visit in the 1 year before the index date (1 562 288 patients) (eFigure 1 in Supplement 1).

### Patient- and Facility-Level Factors

The 23-year study period was categorized into index date groups of 2000 to 2005, 2006 to 2011, 2012 to 2017, and 2018 to 2022. Information was obtained from data available on or before the index date (detailed definitions in eTable 1 in Supplement 1). We collected demographics (eg, age) and social-behavioral demographics (eg, income). Because guideline-recommended antihypertensive treatment varies based on patient race according to available evidence,^6,14^ we evaluated race and ethnicity as factors associated with β-blocker treatment. Data on race and ethnicity were self-reported based on enrollment files wherein the veteran self-reported their race and ethnicity, further categorized for this analysis as Hispanic, Non-Hispanic Black, Non-Hispanic White, Non-Hispanic other (including Asian American, American Indian or Alaskan Native, and Native Hawaiian or other Pacific Islander), or unknown or missing. We selected the most recent value in the 1 year period before the index date for anthropometric (eg, body mass index) and laboratory measurements (eg, estimated glomerular filtration rate calculated from measured serum creatinine using the CKD-Epi 2021 equation^24^). We averaged all available BP measurements from select outpatient settings in the 6-month period before the index date. Medical diagnoses were defined using validated algorithms from all available claims before the index date (eg, history of CVD),^25,26,27,28,29,30,31,32,33,34,35^ which also enabled classification of each veteran as frail or nonfrail using a validated VA frailty index score.^32,36,37,38^ Pharmacy fill data defined use of aspirin and statin. Finally, we collected the number of primary care visits, hospitalizations, and emergency department visits in the 365 days before the index date. Facility-level information included the Veterans Integrated Service Network (VISN) in which the veteran was receiving care (Northeast, Southeast, Continental, and Pacific)^39^ and receipt of care at a facility defined as (1) academic vs nonacademic and (2) urban vs rural based on designations by the VA’s Office of Rural Health and Area Health resource files.

### Presence or Absence of Compelling Indications

From an initial list of over 50 cardiac and noncardiac conditions for which β-blockers can be used,^40^ we identified 7 compelling indications for β-blocker use based on guideline recommendations and clinical relevance, including aortic aneurysm and/or disease, angina, atrial fibrillation and/or arrhythmia, chronic liver disease and/or cirrhosis, heart failure with reduced ejection fraction, myocardial infarction, or coronary revascularization.^14,18^ Other diagnoses with insufficient prevalence (<2%) were not included in the final analysis, except for a post hoc sensitivity analysis including migraine as a compelling indication.

### Statistical Analysis

All analyses were completed stratified by presence or absence of compelling indications. We first summarized baseline characteristics overall and among those with and without compelling indications, separately. We then evaluated the trends in the proportion initiating β-blocker regimens across calendar periods by modeling β-blocker use as a dependent variable and the index date periods as a categorical independent variable. Because the β-blocker class is heterogenous and evidence supporting use of specific β-blockers has changed over time, we further descriptively assessed trends for atenolol, carvedilol, metoprolol (succinate or tartrate), and propranolol.

We assessed factors associated with β-blocker initiation among patients without compelling indications, as we were most interested in clinical factors associated with inappropriate β-blocker use in this population. Multivariate Poisson regression models with robust standard errors generated prevalence ratios (PR) and 95% CIs. We constructed 3 nested models. Model 1 was unadjusted (ie, crude). Model 2 was age- and sex- adjusted. Model 3 included adjustment for all variables in model 2 and all characteristics listed in Table 1. Model fit was assessed using pseudo-R^2^, calculated as 1–(deviance/null deviance), and the deviance statistic. Given that patients with missing data were inherently different from those who were not missing data (eTables 2-3 in Supplement 1), patients with missing data on any of the model variables were excluded from that analysis. These analyses were repeated by index year (2000-2014 and 2015-2022 analyzed separately) because the publication of JNC-7 encouraging β-blocker use second line occurred in late 2014. To evaluate potential multicollinearity, we generated a correlation matrix that accommodated mixed data types. None of the 903 pairwise comparisons across 43 variables met a threshold of |*r*| > 0.90, which would indicate severe collinearity (eFigure 2 in Supplement 1).^41,42^ All analyses were completed using R version 4.1.2 (R Project for Statistical Computing).

**Table 1.** Characteristics of Veterans Initiating Antihypertensive Medications in the Veterans Health Administration for Incident Treated Hypertension, 2000 Through 2022

Variable	Patients, No. (%)		
	Overall (N = 3 138 304)	Compelling indication for β-blocker	
		Yes (n = 223 482)	No (n = 2 914 822)
Index y category			
2000-2005	960 231 (30.6)	56 814 (25.4)	903 417 (31.0)
2006-2011	914 552 (29.1)	60 046 (26.9)	854 506 (29.3)
2012-2017	721 793 (23.0)	53 678 (24.0)	668 115 (22.9)
2018-2022	541 728 (17.3)	52 944 (23.7)	488 784 (16.8)
Demographics			
Age, mean (SD), y	61 (13.0)	65 (12.0)	61 (13.0)
Sex			
Male	2 958 488 (94.3)	215 363 (96.4)	2 743 125 (94.1)
Female	179 816 (5.7)	8119 (3.6)	171 697 (5.9)
Race and ethnicity			
Hispanic	149 807 (4.8)	9267 (4.1)	140 540 (4.8)
Non-Hispanic Black	490 636 (15.6)	29 434 (13.2)	461 202 (15.8)
Non-Hispanic White	2 028 127 (64.6)	151 840 (67.9)	1 876 287 (64.4)
Other^a^	66 798 (2.1)	4010 (1.8)	62 788 (2.2)
Unknown/missing	402 936 (12.8)	28 931 (12.9)	374 005 (12.8)
Social and behavioral			
County-level per-capita income ($US), mean (SD)^b^	42 381 (124 879.1)	40 594 (132 828.3)	42 522 (124 226.2)
Priority group status			
1 (highest need)	477 060 (15.2)	37 770 (16.9)	439 290 (15.1)
2 through 8	2 073 034 (66.1)	155 914 (69.8)	1 917 120 (65.8)
Unknown/missing	588 210 (18.7)	29 798 (13.3)	558 412 (19.2)
Current smoker	516 461 (16.5)	52 540 (23.5)	463 921 (15.9)
Homeless or history of homelessness	89 114 (2.8)	9340 (4.2)	79 774 (2.7)
Clinical/laboratory measurements			
Body mass index, mean (SD)^c^	30 (5.9)	30 (6.1)	30 (5.9)
Systolic blood pressure category (mmHg)			
<140	1 244 872 (44.3)	109 940 (55.7)	1 086 904 (41.5)
140 to <160	1 076 704 (38.3)	62 940 (31.9)	1 039 516 (39.7)
≥160	489 381 (17.4)	24 487 (12.4)	491 806 (18.8)
Diastolic blood pressure category (mmHg)			
<80	1 099 709 (39.1)	102 048 (51.7)	986 564 (37.7)
80 to <100	1 465 325 (52.1)	86 098 (43.6)	1 400 517 (53.5)
≥100	245 923 (8.7)	9221 (4.7)	231 145 (8.8)
Heart rate, mean (SD), beats per minute	76 (13.8)	75 (14.4)	76 (13.8)
Total cholesterol, median (IQR), mg/dL	184 (157.0-213)	170 (143.0-200)	185 (158.0-214)
High-density lipoprotein cholesterol, median (IQR), mg/dL	43 (36.0-52)	42 (35.0-51)	43 (36.0-52)
Low-density lipoprotein cholesterol, median (IQR), mg/dL	109 (85.4-134)	98 (76.0-124)	110 (86.0-135)
Triglycerides, median (IQR), mg/dL	131 (90.0-196)	122 (84.0-181)	132 (90.0-198)
Hemoglobin A_1c_, median (IQR), %	6 (5.6-7)	6 (5.6-7)	6 (5.5-7)
Estimated glomerular filtration rate, mean (SD), mL/min/1.73m^2^	83 (19.5)	79 (21.5)	83 (19.3)
Medical conditions			
Alcohol use disorder	100 702 (3.2)	16 821 (7.5)	83 881 (2.9)
Aortic aneurysm/disease	9730 (0.3)	9730 (4.4)	0
Angina	10 203 (0.3)	10 203 (4.6)	0
Atrial fibrillation or arrhythmia	64 625 (2.1)	64 625 (28.9)	0
Chronic kidney disease	61 939 (2.0)	17 778 (8.0)	44 161 (1.5)
Chronic liver disease or cirrhosis	34 877 (1.1)	34 877 (15.6)	0
Diabetes	332 975 (10.6)	43 887 (19.6)	289 088 (9.9)
Depression	273 502 (8.7)	28 344 (12.7)	245 158 (8.4)
Drug/substance use	71 809 (2.3)	14 450 (6.5)	57 359 (2.0)
End-stage kidney disease or dialysis	731	354 (0.2)	377
Frail based on the VA frailty index^d^	67 378 (2.1)	22 558 (10.1)	44 820 (1.5)
Heart failure with reduced ejection fraction	120 832 (3.9)	120 832 (54.1)	0
Migraine	37 551 (1.2)	1675 (0.7)	35 876 (1.2)
Myocardial infarction	9979 (0.3)	9979 (4.5)	0
Obstructive sleep apnea	99 395 (3.2)	14 882 (6.7)	84 513 (2.9)
Peripheral artery disease	28 946 (0.9)	8310 (3.7)	20 636 (0.7)
Prior coronary revascularization	3758 (0.1)	3758 (1.7)	0
Stroke	111 259 (3.5)	15 735 (7.0)	95 524 (3.3)
Medication use			
Aspirin	308 438 (9.8)	43 616 (19.5)	264 822 (9.1)
Statin	1 054 614 (33.6)	89 432 (40.0)	965 182 (33.1)
Health care utilization in prior y			
Primary care visits, median [IQR]	2 (1.0-4.0)	4.0 (2.0-8)	2.0 (1.0-4.0)
≥1 Hospitalization	190 961 (6.1)	62 106 (27.8)	128 855 (4.4)
≥1 Emergency department visit	473 026 (15.1)	65 225 (29.2)	407 801 (14.0)
VISN Region^e^			
Northeast	954 488 (30.4)	77 775 (34.8)	876 713 (30.1)
Southeast	1 028 113 (32.8)	68 044 (30.4)	960 069 (32.9)
Continental	653 126 (20.8)	44 198 (19.8)	608 928 (20.9)
Pacific	502 577 (16.0)	33 465 (15.0)	469 112 (16.1)
Academic setting	2 090 117 (66.6)	159 111 (71.2)	1 931 006 (66.2)
Urban setting	169 060 (5.4)	10 041 (4.5)	159 019 (5.5)

Abbreviations: ED, emergency department; VISN, Veterans Integrated Service Network.

SI conversion factors: To convert hemoglobin A_1c_ to proportion of total hemoglobin, multiply by 0.01; high-density lipoprotein to mmol/L, multiply by 0.0259; low-density lipoprotein to mmol/L, multiply by 0.0259; total cholesterol to mmol/L, multiply by 0.0259; triglycerides to mmol/L, multiply by 0.0113.

^a^ Includes Asian American, American Indian or Alaskan Native, and Native Hawaiian or Other Pacific Islander.

^b^ According to the US Bureau of Economic Analysis, matched to zip or Federal Information Processing Series code.

^c^ Body mass index is calculated as weight in kilograms divided by height in meters squared.

^d^ Ratio of the sum of the number of health deficits relative to 31 health factors evaluated, ranging from 0 to 1, with 0 indicating no frailty and non-0 numbers indicating some degree of frailty. Nonfrail was defined as a calculated frailty index 0.21 or less, and frail was defined as a calculated frailty index higher than 0.21.

^e^ Categorized according to the Department of Veterans Affairs regional offices map. The Northeast region was composed of VISNs 1, 2, 4, 5, 10, and 12. The Southeast region was composed of VISNs 6, 7, 8, 9, and 16. The Continental region consisted of VISNs 15, 17, 18, 19, and 23. Finally, the Pacific region was composed of VISNs 20, 21, and 22.

## Results

### Baseline Characteristics

Among the 3 138 304 veterans included in the cohort, the mean (SD) age was 61.0 (13.0) years, 2 958 488 (94.3%) were male, and 149 807 (4.8%) were Hispanic, 490 636 (15.6%) were Non-Hispanic Black, 2 028 127 (64.6%) were non-Hispanic White, and 66 798 (2.1%) were other races and ethnicities (Table 1). The vast majority of the overall cohort (2 914 822 patients [92.9%]) did not have a compelling indication for a β-blocker. Veterans with vs without compelling indications were more likely to be older, current smokers, and have systolic BP less than 140 mm Hg or diastolic BP less than 80 mm Hg. All concomitant medical diagnoses were more prevalent among patients with vs without compelling indications. Veterans with compelling indications were more likely to use aspirin or statins.

### β-Blocker Use and Compelling Indication Status

**Figure 1.**
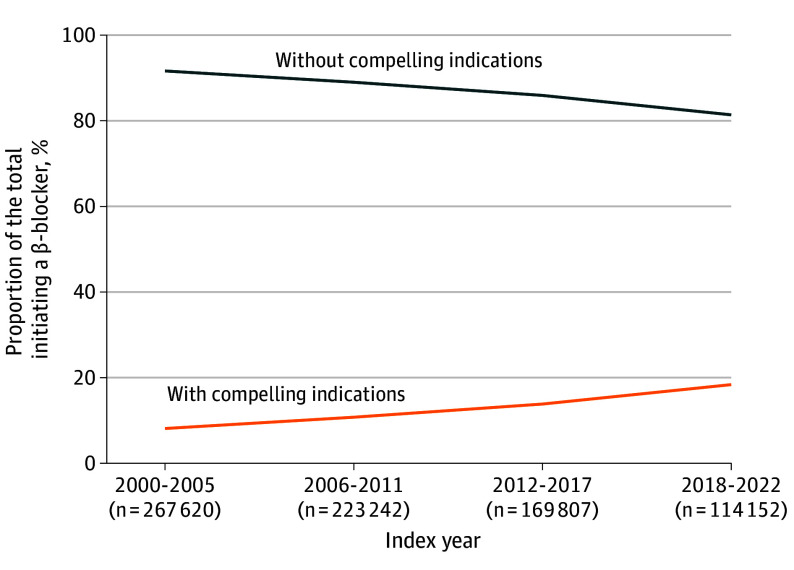
Compelling Indication Status Among Veterans Who Initiated a β-Blocker for Hypertension Treatment, 2000 Through 2022

Overall, 774 821 of 3 138 304 veterans (24.7%) initiated a β-blocker; of these, 90 776 (11.7%) had a compelling indication and 684 045 (88.3%) did not. β-blockers were most commonly initiated as monotherapy (372 330 of 774 821 [48.1%]) or in combination with other first-line classes (373 882 of 774 821 [48.3%]) (eTable 4 in Supplement 1). The proportion of veterans initiating β-blockers who did not have a compelling indication decreased from 91.8% in 2000 through 2005 (245 703 of 267 620) to 81.5% in 2018 to 2022 (93 088 of 114 152) (Figure 1).

Of the 223 482 veterans with a compelling indication overall, 90 776 (40.6%) initiated a β-blocker, which increased from 21 917 (38.6%) in 2000 through 2005 to 24 175 (40.3%) in 2006 to 2011 and 23 620 (44.0%) in 2012 to 2017, then declined to 21 064 (39.8%) in 2018 to 2022 (Table 2). Among the 2 914 822 veterans without a compelling indication overall, 684 045 (23.5%) initiated a β-blocker, which decreased steadily each year from 245 703 (27.2%) in 2000 through 2005 to 93 088 (19.0%) in 2018 to 2022.

**Table 2.** Initiation of β-Blocker Regimens and Specific β-Blockers Initiated Among Veterans With Incident Hypertension, 2000-2022

Population and medication regimen	Patients, No. (%)				
	Overall	Index year			
		2000-2005	2006-2011	2012-2017	2018-2022
With compelling indications					
No.	223 482	56 814	60 046	53 678	52 944
Non–β-blocker regimen initiated	132 706 (59.4)	34 897 (61.4)	35 871 (59.7)	30 058 (56.0)	31 880 (60.2)
β-blocker regimen initiated	90 776 (40.6)	21 917 (38.6)	24 175 (40.3)	23 620 (44.0)	21 064 (39.8)
Atenolol^a^	12 657 (13.9)	6599 (30.1)	3427 (14.2)	1762 (7.5)	869 (4.1)
Carvedilol^a^	19 453 (21.4)	2578 (11.8)	4964 (20.5)	6602 (28.0)	5309 (25.2)
Metoprolol^a^	54 998 (60.6)	11 959 (54.6)	14 849 (61.4)	14 279 (60.5)	13 911 (66.0)
Propranolol^a^	2756 (3.0)	610 (2.8)	734 (3.0)	722 (3.1)	690 (3.3)
Other β-blocker^a^	1015 (1.1)	201 (0.9)	236 (1.0)	275 (1.2)	303 (1.4)
Without compelling indications					
No.	2 914 822	903 417	854 506	668 115	488 784
Non–β-blocker regimen initiated	2 230 777 (76.5)	657 714 (72.8)	655 439 (76.7)	521 928 (78.1)	395 696 (81.0)
β-blocker regimen initiated	684 045 (23.5)	245 703 (27.2)	199 067 (23.3)	146 187 (21.9)	93 088 (19.0)
Atenolol^a^	234 543 (34.3)	123 424 (50.2)	68 781 (34.6)	31 469 (21.5)	10 869 (11.7)
Carvedilol^a^	57 181 (8.4)	5836 (2.4)	13 524 (6.8)	22 016 (15.1)	15 805 (17.0)
Metoprolol^a^	348 583 (51.0)	105 582 (43.0)	107 065 (53.8)	81 140 (55.5)	54 796 (58.9)
Propranolol^a^	35 819 (5.2)	8466 (3.4)	7957 (4.0)	9756 (6.7)	9640 (10.4)
Other β-blocker^a^	8453 (1.2)	2577 (1.0)	1903 (1.0)	1927 (1.3)	2046 (2.2)

^a^ Proportions are relative to the total initiating a β-blocker, expressed as a percentage.

### Use of Specific β-Blockers

From 2000 through 2005 to 2018 to 2022, metoprolol initiation increased (with compelling indications: 11 959 of 21 917 [54.6%] to 13 911 of 21 064 [66.0%]; without compelling indications: 105 582 of 245 703 [43.0%] to 54 796 of 93 088 [58.9%]) (Table 2). Similarly, carvedilol initiation increased (with compelling indications: 2578 of 21 917 [11.8%] to 5309 of 21 064 [25.2%]; without compelling indications: 5836 of 245 703 [2.4%] to 15 805 of 93 088 [17.0%]). Atenolol initiation decreased dramatically (with compelling indications: 6599 of 21 917 [30.1%] to 869 of 21 064 [4.1%]; without compelling indications: 123 424 of 245 703 [50.2%] to 10 869 of 93 088 [11.7%]). Propranolol initiation was relatively stable over time among those with compelling indications (610 of 21 917 [2.8%] to 690 of 21 064 [3.3%]) but increased dramatically in patients without compelling indications (8466 of 245 703 [3.4%] to 9640 of 93 088 [10.4%]).

### Factors Associated With β-Blocker Initiation Without Compelling Indications

**Figure 2.**
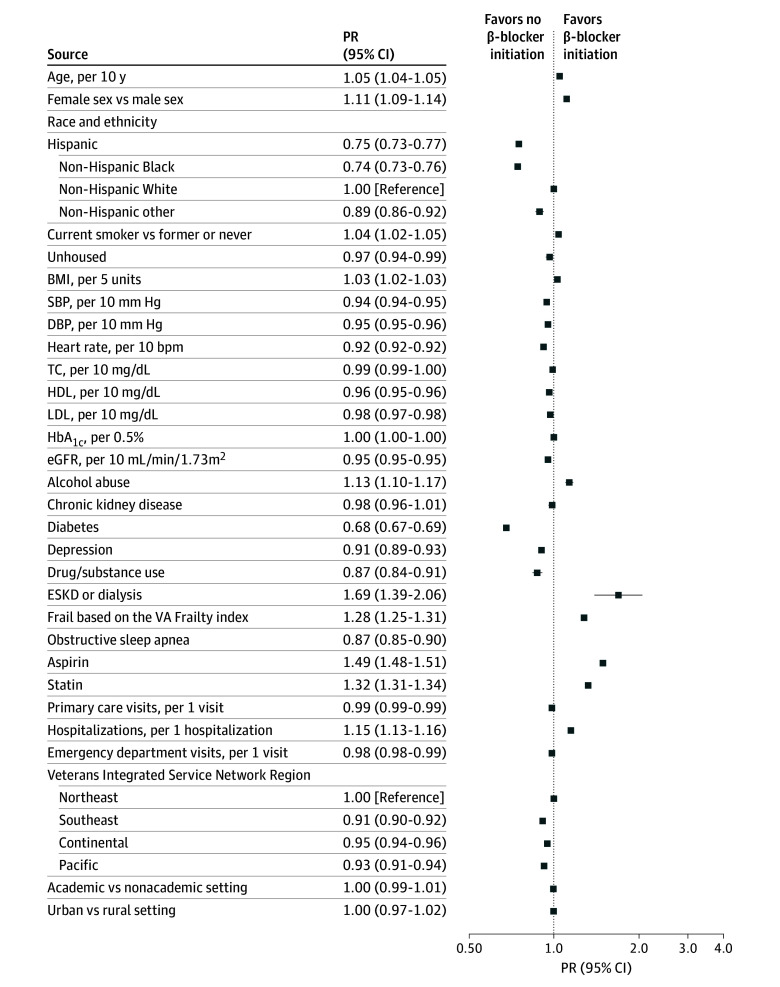
Fully Adjusted Prevalence Ratios (PRs) for Factors Associated With β-Blocker Initiation Prevalence ratios are results from model 3, which are adjusted for all sociodemographic, laboratory, medical condition, medication use, and utilization data shown in Table 1. To convert hemoglobin A_1c_ to proportion of total hemoglobin, multiply by 0.01; high-density lipoprotein to mmol/L, multiply by 0.0259; low-density lipoprotein to mmol/L, multiply by 0.0259; total cholesterol to mmol/L, multiply by 0.0259. BMI indicates body mass index; DBP, diastolic blood pressure; eGFR, estimated glomerular filtration rate; ESKD, end-stage kidney disease; HbA_1c_, glycated hemoglobin; HDL, high-density lipoprotein cholesterol; LDL, low-density lipoprotein cholesterol; SBP, systolic blood pressure; TC, total cholesterol; VA, Veterans Health Administration.

PRs and associated 95% CIs for the unadjusted (model 1), age- and sex-adjusted (model 2), and fully adjusted (model 3) results are shown in eTable 5 in Supplement 1. In the fully adjusted model (Figure 2), factors more prevalent among β-blocker initiators were 10-year age increases (PR, 1.05; 95% CI, 1.04-1.05), female sex (PR, 1.11; 95% CI, 1.09-1.14), non-Hispanic White race and ethnicity (Hispanic PR, 0.75; 95% CI, 0.73-0.77; Non-Hispanic Black PR. 0.74; 95% CI, 0.73-0.76; Non-Hispanic other PR, 0.89; 95% CI, 0.86-0.92), and current smoking (PR, 1.04; 95% CI, 1.02-1.05). Compared with individuals who initiated antihypertensive treatment in 2000 through 2005, those initiating in 2006 to 2011 and 2012 to 2017 had similar likelihoods of initiating a β-blocker (PR, 0.99; 95% CI, 0.98-1.01 and PR, 1.02; 95% CI, 1.01-1.04, respectively), and those initiating treatment in 2018 to 2022 had a lower likelihood of initiating a β-blocker (PR, 0.91; 95% CI, 0.90-0.93).

β-blocker initiation was more prevalent with each 5-unit increase in body mass index (calculated as weight in kilograms divided by height in meters squared; PR, 1.03; 95% CI, 1.02-1.03), and initiation was less prevalent with each 10 mL/min/1.73m^2^ increase in eGFR (PR, 0.95; 95% CI, 0.95-0.95). Increasing levels of heart rate and systolic or diastolic BP were associated with reduced likelihood of β-blocker initiation. Medical conditions that were more prevalent among β-blocker initiators included alcohol use disorder (PR, 1.13; 95% CI, 1.10-1.17), end-stage kidney disease or dialysis (PR, 1.68; 95% CI, 1.39-2.06), frailty (PR, 1.28; 95% CI, 1.25-1.31), peripheral artery disease (PR, 1.12; 95% CI, 1.07-1.16), and stroke (PR, 1.15; 95% CI, 1.13-1.18). Aspirin and statin use were associated with increased likelihood of β-blocker initiation (PR, 1.49; 95% CI, 1.49-1.51 and PR, 1.32; 95% CI, 1.31-1.34). Each hospitalization in the 1-year before the index date increased the likelihood of β-blocker initiation (PR, 1.15; 95% CI, 1.13-1.16). Compared with veterans in the Northeast VISN region, those in the Southeast, Continental, and Pacific regions were all less likely to initiate β-blockers (PR, 0.91; 95% CI, 0.90-0.92; PR, 0.95; 95% CI, 0.94-0.96; and PR, 0.93; 95% CI, 0.91-0.94, respectively).

### Secondary and Sensitivity Analyses

Incorporating baseline migraine history as a compelling indication (1.2% of the overall population) did not significantly alter the primary findings, although propranolol was initiated to a greater degree in this sensitivity analysis vs the primary analysis (7.1% vs 3.0%) (eTable 6, eFigure 3 in Supplement 1). In veterans initiating hypertension treatment without compelling indications between 2000 and 2014 and between 2015 and 2022, factors associated with β-blocker use were similar to the primary analysis (eTable 7 in Supplement 1).

## Discussion

This serial cross-sectional study of over 3 million veterans initiating treatment for incident hypertension has 3 main takeaways. First, 88.3% of patients initiating β-blockers did not have a compelling indication, and although this proportion decreased over time, it is nonetheless high at 81.5% in the most recent years of the study. Second, initiation of atenolol has decreased in favor of initiating metoprolol or carvedilol, with significant increases in propranolol use among those without compelling indications. Finally, in adjusted models, factors that were associated with increased likelihood of β-blocker initiation in the absence of compelling indications included older age, female, race and ethnicity, current smoking status, presence of frailty, individuals who were hospitalized in the year before the index date, and receiving care in urban settings or the Northeast US region. Taken together, these findings identify target populations for improving the initial prescribing of antihypertensive regimens to optimize cardiovascular outcomes among US veterans.

Compared with first-line agents, β-blockers represent the most pharmacodynamically heterogenous antihypertensive medication class. Individual β-blockers vary by β-1 receptor selectivity, intrinsic sympathomimetic activity, and peripheral vasodilation via alpha-1 blockade or nitric oxide release.^43,44,45^ Atenolol, a β-1 selective antagonist without intrinsic sympathomimetic activity or vasodilation properties, was the β-blocker used in many early trials comparing β-blockers with placebo or other antihypertensives.^46,47^ These pivotal trials determined that β-blockers were superior to placebo to prevent CVD but inferior to thiazides, CCBs, or ARBs to prevent CVD, specifically stroke and all-cause mortality.^12,13,48,49^ These data, supported by subsequent meta-analyses, formed the basis for not recommending β-blockers as a first-line class due to both adverse effects (eg, fatigue and bradyarrhythmias) and inferior CVD clinical outcomes.^7,10,11,12,13,41,42,50,51^ Some argue that atenolol’s shorter duration of action and once-daily dosing in these studies contributed to suboptimal BP-lowering, consequently leading to inferior stroke protection, although comparative data with other β-blockers are inconclusive.^46,52,53^ Nevertheless, the lack of efficacy was generalized to the class, despite the potential that β-blocker efficacy for essential hypertension may not be a class effect—similar to their use in heart failure with reduced ejection fraction, where only metoprolol succinate, bisoprolol, and carvedilol have demonstrated outcome benefits.^54,55,56^ The 2023 ESH BP guideline takes a different stance by supporting β-blockers as first-line agents in part due to their broad utility in treating other cardiac and noncardiac conditions, facilitating individualized treatment.^57^ However, the observed high rate of β-blocker use without compelling indications suggests that prescribing patterns are driven by more than individualized treatment.

The current analysis observed prescribing differences according to age, history of alcohol use disorder, chronic kidney disease or end-stage kidney disease and/or dialysis, depression, diabetes, frailty, and obstructive sleep apnea. These findings underscore the need to prioritize guideline-recommended first-line agents and to critically evaluate β-blocker prescribing in the absence of clear clinical indications, which may expose patients to avoidable harm without comparable benefit. Over the 23-year study period, we found marked reductions in atenolol initiation alongside increases in metoprolol, carvedilol, and propranolol. As recommended by the 2023 ESH BP guidelines and others,^18,51^ additional studies are needed to compare newer β-blockers with one another and atenolol for uncomplicated hypertension.

In 2 observational studies of Medicare and Medicaid beneficiaries with and without compelling indications, 11% to 36% of patients with newly-diagnosed hypertension initiated a β-blocker, which is unchanging over time or slightly decreasing after the release of JNC-8, similar to our findings.^58,59,60^ One study similarly observed regional differences in likelihood of β-blocker initiation and lower likelihood of β-blocker monotherapy initiation in Black vs White and male vs female patients.^60^ Geographic variations in prescribing and treatment are common across chronic conditions and health systems, and additional multilevel modeling analyses may be needed to understand the 5% to 9% variation in β-blocker prescribing across geographic regions observed in the current analysis. Our study extends the literature by examining predictors of β-blocker initiation stratified by compelling indications and incorporates the most recent data through 2022, including the COVID-19 era.

### Strengths and Limitations

The current analysis is strengthened by using medication dispensing data and linking data to clinical, claims, and enrollment information to capture social characteristics, vital signs, medical conditions, and health care utilization metrics. The 23-year study period allowed us to evaluate trends in clinical practice medication use and provide broad historical context. Nonetheless, several important limitations should be noted. The first is that patients of the VA are predominantly male and non-Hispanic White, and these findings should be cautiously applied to populations poorly represented in this analysis. Similarly, our results may not generalize to the entire veteran population, as we were not able to assess external health care use or prescription dispenses. Medical conditions were identified using claims data and validated algorithms where possible, though shifts in coding practices over time and across regions may have influenced estimates. Given the hypothesis-generating nature of these analyses, some statistically significant findings may reflect chance associations, as we included a large number of covariates without formal corrections for multiple comparisons. We focused on 7 compelling indications for β-blocker use, excluding rarer conditions like essential tremor due to low prevalence and coding ambiguity. A sensitivity analysis excluding patients with migraine showed minimal impact on findings, though future work could examine additional secondary indications. Missingness in key variables may have affected the fully adjusted models, and while we adjusted for a wide range of covariates, unmeasured confounding—particularly related to clinical decision-making—remains possible. Additionally, use of dispensing data to identify aspirin use may have poor sensitivity, indicating that patients truly taking aspirin may not be detected in the data and increasing the likelihood for misclassification, although this approach minimizes false positives and has high specificity.^61^

## Conclusions

In this cross-sectional study, β-blockers were often prescribed for veterans without compelling indications at the initial point of hypertension treatment from 2000 through 2022. Efforts are needed to align β-blocker prescribing with clinical guidelines for patients with and without compelling indications. Additional research may focus on clinical outcomes associated with the patterns described herein, particularly stroke, overall and among specific β-blocker classes.

## Notes

Supplement 1. **eMethods.** Data Sources **eFigure 1.** Flowchart for Inclusion in the Current Analysis **eFigure 2.** Correlation Coefficient Heat Map Among 43 Covariates (903 Pairwise Comparisons) **eFigure 3.** Fully Adjusted Prevalence Ratios (Model 3) for Factors Associated With β-Blocker Initiation, Sensitivity Analysis Including Migraine as a Compelling Indication **eTable 1.** Variable Definitions **eTable 2.** Missingness Table, Overall and by Compelling Indication Status **eTable 3.** Characteristics of Those Missing vs Not Missing ≥1 Key Variables in the Fully Adjusted Analysis, Overall and by Compelling Indication Status **eTable 4. **Initiation of Specific β-Blocker Regimens **eTable 5.** Prevalence Ratios and 95% CIs for Factors Associated With Initiation of β-Blockers Among Those Without Compelling Indications **eTable 6. **Initiation of β-Blocker Regimens and Specific β-Blockers Initiated Among Veterans With Incident Hypertension, 2000-2022, Including Migraine as a Compelling Indication **eTable 7.** Sensitivity Analysis Evaluating Factors Associated With Initiation of β-Blockers for Veterans Starting Antihypertensive Medications January 1, 2000, Through December 31, 2014, and January 1, 2015, Through December 31, 2022

Supplement 2.
Data Sharing Statement
