# Supplementary material for: First-Line β-Blocker Use for Hypertension in the Veterans Health Administration
Source: JAMA Netw Open. 2025 Aug 27;8(8):e2529026. doi: 10.1001/jamanetworkopen.2025.29026 (PMC12391979; doi:10.1001/jamanetworkopen.2025.29026)
Supplement: Supplement 1. — eMethods. Data Sources eFigure 1. Flowchart for Inclusion in the Current Analysis eFigure 2. Correlation Coefficient Heat Map Among 43 Covariates (903 Pairwise Comparisons) eFigure 3. Fully Adjusted Prevalence Ratios (Model 3) for Factors Associated With β-Blocker Initiation, Sensitivity Analysis Including Migraine as a Compelling Indication eTable 1. Variable Definitions eTable 2. Missingness Table, Overall and by Compelling Indication Status eTable 3. Characteristics of Those Missing vs Not Missing ≥1 Key Variables in the Fully Adjusted Analysis, Overall and by Compelling Indication Status eTable 4. Initiation of Specific β-Blocker Regimens eTable 5. Prevalence Ratios and 95% CIs for Factors Associated With Initiation of β-Blockers Among Those Without Compelling Indications eTable 6. Initiation of β-Blocker Regimens and Specific β-Blockers Initiated Among Veterans With Incident Hypertension, 2000-2022, Including Migraine as a Compelling Indication eTable 7. Sensitivity Analysis Evaluating Factors Associated With Initiation of β-Blockers for Veterans Starting Antihypertensive Medications January 1, 2000, Through December 31, 2014, and January 1, 2015, Through December 31, 2022 [file jamanetwopen-e2529026-s001.pdf]

Supplemental Online Content

Derington CG, Berchie RO, Mohanty AF, et al. First-line β-blocker use in the Veterans Health Administration. *JAMA Netw Open*. 2025;8(8):e2529026. doi:10.1001/jamanetworkopen.2025.29026

eMethods. Data Sources
eFigure 1. Flowchart for Inclusion in the Current Analysis
eFigure 2. Correlation Coefficient Heat Map Among 43 Covariates (903 Pairwise Comparisons)
eFigure 3. Fully Adjusted Prevalence Ratios (Model 3) for Factors Associated With β-Blocker Initiation, Sensitivity Analysis Including Migraine as a Compelling Indication
eTable 1. Variable Definitions
eTable 2. Missingness Table, Overall and by Compelling Indication Status
eTable 3. Characteristics of Those Missing vs Not Missing ≥1 Key Variables in the Fully Adjusted Analysis, Overall and by Compelling Indication Status
eTable 4. Initiation of Specific β-Blocker Regimens
eTable 5. Prevalence Ratios and 95% CIs for Factors Associated With Initiation of β-Blockers Among Those Without Compelling Indications
eTable 6. Initiation of β-Blocker Regimens and Specific β-Blockers Initiated Among Veterans With Incident Hypertension, 2000-2022, Including Migraine as a Compelling Indication
eTable 7. Sensitivity Analysis Evaluating Factors Associated With Initiation of β-Blockers for Veterans Starting Antihypertensive Medications January 1, 2000, Through December 31, 2014, and January 1, 2015, Through December 31, 2022

This supplemental material has been provided by the authors to give readers additional information about their work.

© 2025 Derington CG et al. *JAMA Network Open*.

eMethods. Data Sources

We obtained demographic, clinical, and healthcare utilization data from the VA's Corporate Data Warehouse and pharmacy data from Managerial Cost Accounting, Pharmacy Benefits Management, and the Corporate Data Warehouse.

© 2025 Derington CG et al. *JAMA Network Open*.

eFigure 1. Flowchart for Inclusion in the Current Analysis

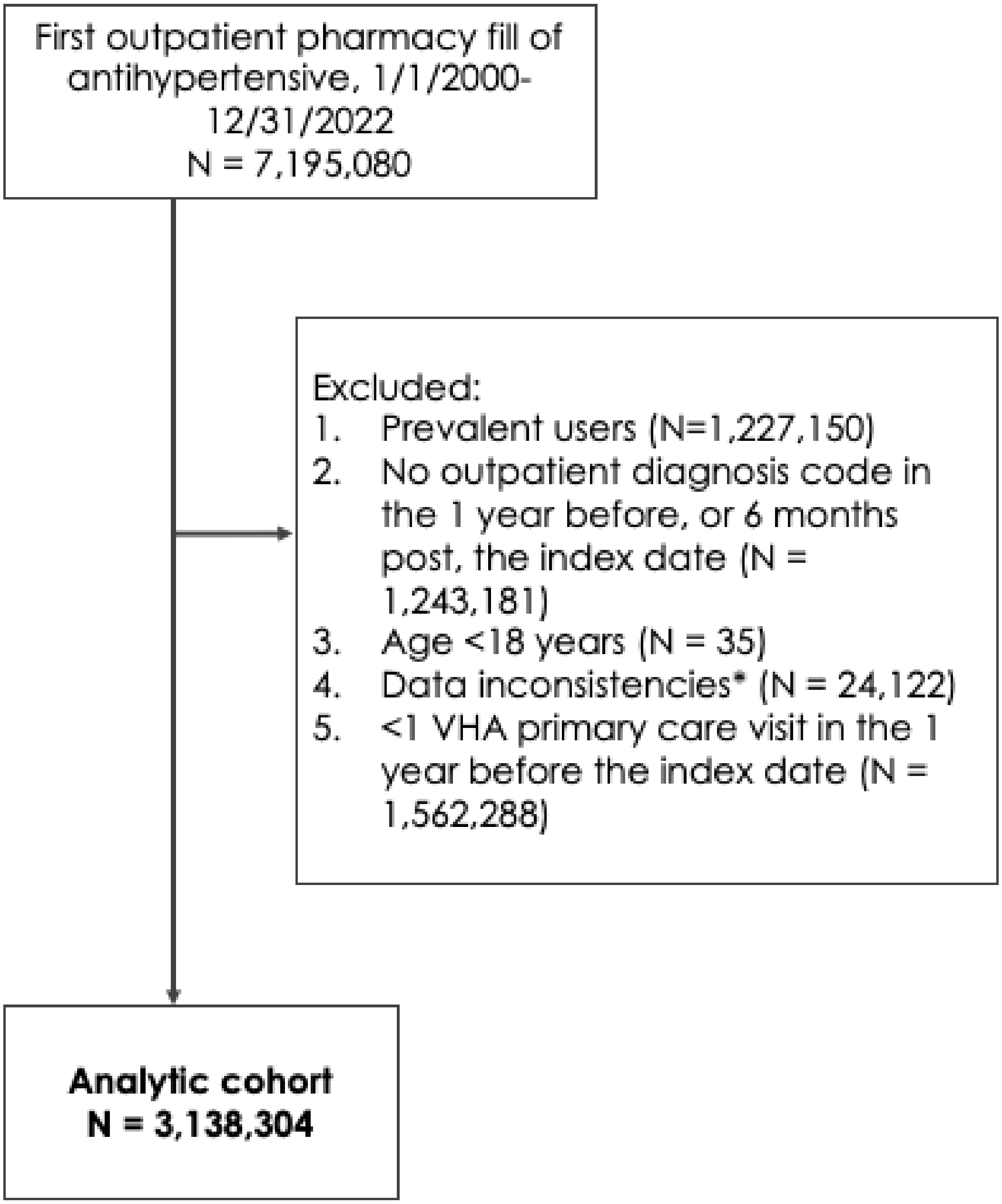

* Data inconsistencies included: test patients, not Veterans, missing dates of birth, death date occurred prior to the index date, multiple death dates, or age >90 years)

Abbreviations: VA: Veterans Affairs

© 2025 Derington CG et al. *JAMA Network Open*.

eFigure 2. Correlation Coefficient Heat Map Among 43 Covariates (903 Pairwise Comparisons)

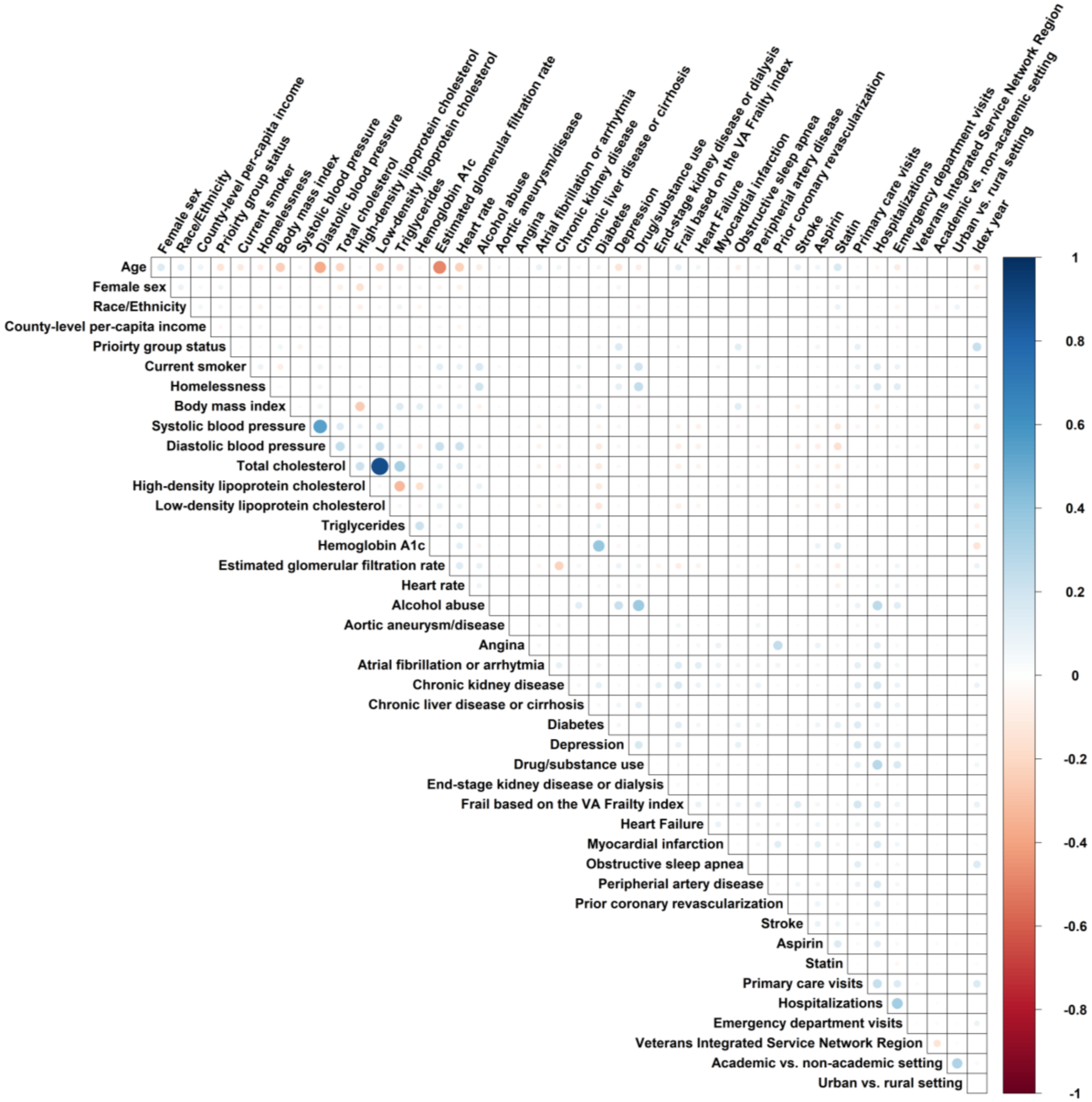

For continuous–continuous variable pairs, we used the Pearson correlation coefficient, which quantifies the linear relationship between two continuous variables. For binary–continuous pairs, we calculated the point-biserial correlation, a special case of the Pearson correlation that measures the association between a dichotomous variable and a continuous variable. For binary–binary pairs, we used Cramér's V, which captures the strength of association between two nominal binary variables based on the chi-squared statistic. These methods allowed us to construct a comprehensive correlation matrix accommodating the mixed types of variables in the dataset.

Correlation coefficients across all 903 pairwise comparisons were small: the median absolute value was 0.03, the 95th-percentile was 0.16, and only two pairs exceeded $|r| = 0.50$. The strongest association was observed between total cholesterol and low-density lipoprotein cholesterol ($r = 0.88$)

© 2025 Derington CG et al. *JAMA Network Open*.

eFigure 3. Fully Adjusted Prevalence Ratios (Model 3) for Factors Associated With β-Blocker Initiation, Sensitivity Analysis Including Migraine as a Compelling Indication

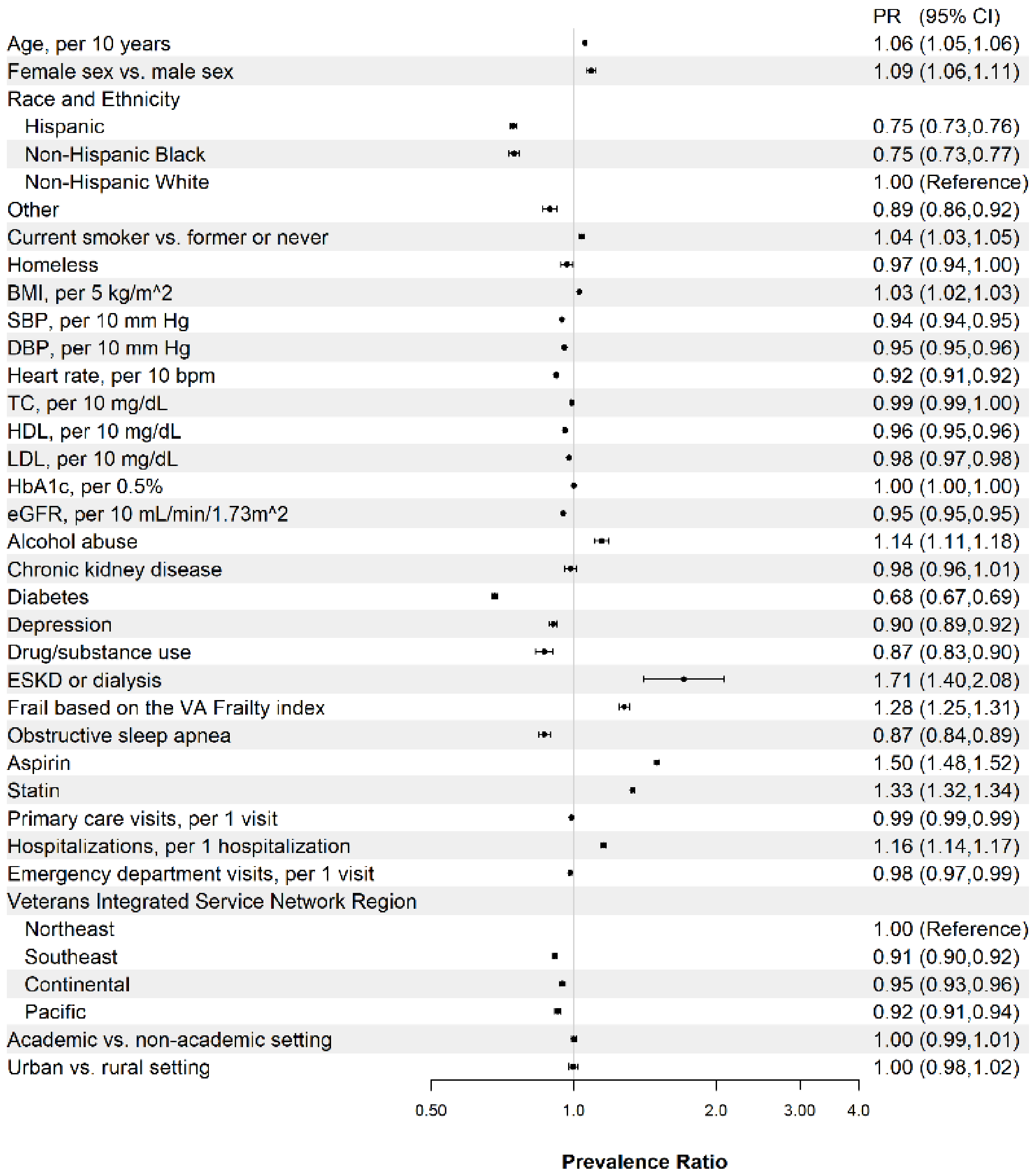

Prevalence ratios are results from model 3, which are adjusted for all socio-demographic, laboratory, medical conditions, medication use, and utilization data shown in Table 1. Point estimates greater than 1 indicate that β-blocker initiation is more likely, whereas prevalence ratios less than 1 indicate that β-blocker initiation is less likely.

Abbreviations: BMI: body mass index; DBP: diastolic blood pressure; eGFR: estimated glomerular filtration rate; ESKD: end-stage kidney disease; HbA1c: hemoglobin A1c; HDL: high-density lipoprotein cholesterol; LDL: low-density lipoprotein cholesterol; SBP: systolic blood pressure; TC: total cholesterol

© 2025 Derington CG et al. *JAMA Network Open*.

eTable 1. Variable Definitions

Variable	Definition
Index date	Date of the first-ever outpatient pharmacy dispense of an antihypertensive medicatino (i.e., new users) in the index date identification period.
Index date identification period	January 1, 2000, to December 31, 2022 (dates inclusive).
Hypertension	Any of the following using all available outpatient claims in the one year prior to, or 180 days after, the index date, including the index date).<br>a) ICD-9 codes: ≥1 outpatient claims with a diagnosis code (any position) of 401.x, 403.0x, 403.1x, 403.9x.<br>b) ICD-10 codes: ≥1 outpatient claims with a diagnosis code (any position) of I10, I12.0, I12.9.
Socio-demographics	
Age	Age of Veterans calculated on the index date based on their date of birth available in the Veteran summary file.
Sex	Male or female
Race	White, Black, Asian, American Indian or Alaskan Native, Native Hawaiian or Other Pacific Islander, Other, and Missing
Ethnicity	Hispanic or non-Hispanic
Race and ethnicity group	Categorized for this analysis as Non-Hispanic White, Non-Hispanic Black, Hispanic, Non-Hispanic Other, and Unknown/Missing. If a patient was missing race or ethnicity, they could not be categorized into a specific race and ethnicity category, and therefore were categorized as "unknown/missing". Non-Hispanic Other includes patients who identified as Non-Hispanic with a race of one of the following: Asian American, American Indian or Alaskan Native, and Native Hawaiian or Other Pacific Islander.
Zip code	Zip code of the Veteran.
FIPS code	Federal Information Processing Standard of the Veteran.
County-level per-capita income	The Veteran's FIPS code was matched to the county-level per-capita income available from the US Bureau of Economic Analysis (https://apps.bea.gov/regional/histdata/index.cfm). The county-level per-capita income value was also matched to the Veteran's index year.
Veterans Integrated Service Network (VISN)	Defined by receipt of care in one of the 23 VISNs. Will be categorized into 4 regions after data query: Northeast, Southeast, Continental, and Pacific according to the VA regional offices map.[4] The Northeast region is comprised of VISNs 1, 2, 4, 5, 10, and 12. The Southeast region is comprised of VISNs 6, 7, 8, 9, and 16. The Continental region is comprised of VISNs 15, 17, 18, 19, and 23. Finally, the Pacific region is comprised of VISNs 20, 21, and 22.

© 2025 Derington CG et al. *JAMA Network Open*.

Supplemental insurance type	Coded as Medicare, Medicaid, private (all insurance external to Medicare/Medicaid), none, and unknown.
Priority group status	Coded as 1 through 9 or multiple.
Current Smoking	Any of the following within one year prior to the index date (including the index date):<br>(a) ICD-9 codes:<br>a. ≥1 hospitalization with a discharge diagnosis code (any position) of tobacco use of 305.1, 649.0x, 989.84, or V15.82) in any discharge position<br>b. ≥1 physician evaluation and management visit with a diagnosis code (any position) of tobacco use of 305.1, 649.0x, 989.84, or V15.82) in any discharge position<br>(b) ICD-10 codes:<br>a. ≥1 hospitalization with a discharge diagnosis code (any position) of tobacco use of F17.200, F17.201, F17.210, F17.211, F17.220, F17.221, F17.290, F17.291, or Z87.891) in any discharge position<br>b. ≥1 outpatient visit with a diagnosis code (any position) of tobacco of F17.200, F17.201, F17.210, F17.211, F17.220, F17.221, F17.290, F17.291, or Z87.891) in any discharge position<br>(c) ≥1 hospitalization with a discharge diagnosis code or physician evaluation and management visit of tobacco use with a CPT code (any position) of 99406, 99407, G0436, G0437, G9016, S9453, S4995, G9276, G9458, 1034F, 4004F, 4001F<br>(d) ≥1 pharmacy claim for nicotine or varenicline in the 379 days before the index date (including the index date).<br>(e) Self-reported smoking status as "current"
Homelessness or history of homelessness[25]	Meets at least one of the following criteria using all available claims prior to the index date (including the index date):<br>a) Claim with at least one of the following outpatient stop codes: 201, 504, 508, 511, 522, 528, 529, 530, 590, 591, 592<br>b) Outpatient claim with at least one of the following diagnosis codes in any position: V60.0, V60.1, V60.89, V60.9, Z59.0, Z59.1, Z59.8, Z59.9<br>c) Inpatient claim with at least one of the following treatment specialty codes (SpecialtyIEN): 28, 29, 37, 39
Vital signs and laboratory measures	
Height	Height (in m) on the index date or the date closest to the index date during the one-year pre-index period. Values ≥48 inches and ≤the 99th percentile will be retained; outliers will be coded as missing.

© 2025 Derington CG et al. *JAMA Network Open*.

Weight	Weight (in kg) on the index date or the date closest to the index date during the one-year pre-index period. Values ≥70 pounds and ≤500 pounds will be retained; outliers will be coded as missing.
Body mass index	Body mass index on or closest to the index date during the one-year pre-index period. Height and weight measurements do not need to be on the same day. This variable will be calculated from height and weight observations in the CDW vital status file as weight (in kilograms) divided by height (in meters) squared. Values ≥10 kg/m^2 and ≤80 kg/m^2 will be retained; outliers will be coded as missing.
Systolic blood pressure	Average of 6-month pre-index systolic BP values corresponding to an outpatient encounter with locations that are associated with clinics for cardiology, renal, primary care/internal medicine/hypertension/clinical pharmacist, endocrinology/diabetes, or cancer. If there are multiple readings on one date, take a mean of the readings. systolic BP values will be dropped if any of the following was true: missing value (either systolic BP or DBP), systolic less than diastolic, systolic >300 mmHg, or systolic <60 mmHg.
Diastolic blood pressure	Average of 6-month pre-index DBP values corresponding to an outpatient encounter with locations that are associated with clinics for cardiology, renal, primary care/internal medicine/hypertension/clinical pharmacist, endocrinology/diabetes, or cancer. If there are multiple readings on one date, take a mean of the readings. DBP values will be dropped if any of the following was true: missing value (either systolic BP or DBP), diastolic greater than systolic, diastolic <30 mmHg, or diastolic >180 mmHg.
Total cholesterol	The total cholesterol value measured in the outpatient setting closest to the index date in the five-year pre-index period (extended from one year due to infrequency of measurement in clinical practice). Defined using OMOPs mapping where LOINC_Mapped is 2093-3 and Topography is (SERUM, PLASMA, BLOOD, SER/PLA, BLOOD*, SER/PLAS, BLOOD., WS-PLASMA, CC SERUM,HIBBING SERUM, MOFH SERUM, OPCC-SERUM,BLOOD VENOUS, LC-SER, SERUM (QUEST)). Values ≥75 mg/dL and ≤500 mg/dL will be retained; outliers will be coded as missing.
HDL-C level	The HDL-C value measured in the outpatient setting closest to the index date in the five-year pre-index period (extended from one year due to infrequency of measurement in clinical practice). Defined using OMOPs mapping where LOINC_Mapped is 2085-9 and Topography is (SERUM, PLASMA, BLOOD, SER/PLA, BLOOD*, SER/PLAS, BLOOD., WS-PLASMA, CC SERUM,HIBBING SERUM, MOFH SERUM, OPCC-SERUM,BLOOD VENOUS, LC-SER, SERUM (QUEST)). Values ≥10mg/dL and ≤200 mg/dL will be retained; outliers will be coded as missing.
LDL-C level	The LDL-C value measured in the outpatient setting closest to the index date in the five-year pre-index period (extended from one year due to infrequency of measurement in clinical practice). Defined using OMOPs mapping where LOINC_Mapped is (13457-7,18262-6,2089-1,2574-2,9346-8) and Topography is (SERUM, PLASMA, BLOOD, SER/PLA, BLOOD*, SER/PLAS, BLOOD., WS-PLASMA, CC SERUM,HIBBING SERUM, MOFH SERUM, OPCC-SERUM,BLOOD VENOUS,

© 2025 Derington CG et al. *JAMA Network Open*.

	LC-SER, SERUM (QUEST)). Values ≥25 mg/dL and ≤250 mg/dL will be retained; outliers will be coded as missing.
Triglyceride level	The triglyceride value measured in the outpatient setting closest to the index date in the five-year pre-index period (extended from one year due to infrequency of measurement in clinical practice). Defined using OMOPs mapping where LOINC_Mapped is 2571-8 and Topography is (SERUM, PLASMA, BLOOD, SER/PLA, BLOOD*, SER/PLAS, BLOOD., WS-PLASMA, CC SERUM,HIBBING SERUM, MOFH SERUM, OPCC-SERUM,BLOOD VENOUS, LC-SER, SERUM (QUEST)). Values ≥20mg/dL and ≤2000 mg/dL will be retained; outliers will be coded as missing.
Hemoglobin A1c	The glycated hemoglobin (i.e., "hemoglobin A1c") value measured in the outpatient setting closest to the index date in the five-year pre-index period (extended from one year due to infrequency of measurement in clinical practice). Defined using OMOPs mapping where LOINC_Mapped is 4548-4 and Topography is (SERUM, PLASMA, BLOOD, SER/PLA, BLOOD*, SER/PLAS, BLOOD., WS-PLASMA, CC SERUM,HIBBING SERUM, MOFH SERUM, OPCC-SERUM,BLOOD VENOUS, LC-SER, SERUM (QUEST)). Values ≥3% and ≤20% will be retained; outliers will be coded as missing.
Serum creatinine	The serum creatinine value measured in the outpatient setting closest to the index date in the one-year pre-index period. Defined using OMOPs mapping where LOINC_mapped is (2160-0, 38483-4, 77140-2, 40248-7, 44784-7) and Topography in (SERUM, PLASMA, BLOOD, SER/PLA, BLOOD*, SER/PLAS, BLOOD., WS-PLASMA, CC SERUM,HIBBING SERUM, MOFH SERUM, OPCC-SERUM,BLOOD VENOUS, LC-SER, SERUM (QUEST)). Values ≥0.2mg/dL and ≤30 mg/dL will be retained; outliers will be coded as missing.
Estimated glomerular filtration rate	The estimated glomerular filtration rate closest to the index date in the one-year pre-index period. This calculation is based on the CKD-EPI 2021 equation that does not include a race coefficient.[6,7] This calculation can be done manually from age, sex, and serum creatinine; we will also pull the variable eGFR_CKD from the CDW. Values ≥1 mL/min/1.73m^2 and ≤200 mL/min/1.73m^2 will be retained; outliers will be coded as missing.
Comorbidities	
Alcohol use disorder	Either two outpatient claims (7 days apart), or one inpatient claim with at least one of the following diagnosis codes in any position using all available claims prior to the index date (including the index date):<br>1) ICD-9 codes: 291, 291.0, 291.1, 291.2, 291.4, 291.5, 291.8, 291.81, 291.82, 291.89, 291.9, 303.00, 303.01, 303.02, 303.03, 303.90, 303.91, 303.92, 303.93, 305.00, 305.01, 305.02, 305.03<br>2) ICD-10 codes: F10.10, F10.120, F10.121, F10.129, F10.14, F10.150, F10.159, F10.180, F10.181, F10.182, F10.188, F10.19, F10.20, F10.21, F10.220, F10.221, F10.229, F10.230, F10.231, F10.232, F10.239, F10.24, F10.250, F10.259, F10.26, F10.27, F10.280, F10.281,

© 2025 Derington CG et al. *JAMA Network Open*.

	F10.282, F10.288, F10.29, F10.920, F10.921, F10.929, F10.94, F10.950, F10.959, F10.96, F10.97, F10.980, F10.981, F10.982, F10.988, F10.99
Angina	Either two outpatient claims (7 days apart), or one inpatient claim with at least one of the following diagnosis codes in any position using all available claims prior to the index date (including the index date):<br>1) ICD-9 codes: 411.0, 411.1, 411.8, 413.0, 413.1, 413.9<br>2) ICD-10 codes: I20.0 I20.1, I20.8, I20.9, I25.110, I25.111, I25.118, I25.119, I25.700, I25.701, I25.708, I25.709, I25.710, I25.711, I25.718, I25.719, I25.720, I25.721, I25.728, I25.729, I25.730, I25.731, I25.738, I25.739, I25.750, I25.751, I25.758, I25.759, I25.760, I25.761, I25.768, I25.769, I25.790, I25.791, I25.798, I25.799
Aortic aneurysm or disease	Either two outpatient claims (7 days apart), or one inpatient claim with at least one of the following diagnosis codes in any position using all available claims prior to the index date (including the index date):<br>1) ICD-9 codes: 441.x<br>2) ICD-10 codes: I71.x
Atrial fibrillation	Either two outpatient claims (7 days apart), or one inpatient claim with at least one of the following diagnosis codes in any position using all available claims prior to the index date (including the index date):<br>1) ICD-9 codes: 427.31<br>2) ICD-10 codes: I48.x
Arrhythmia	Either two outpatient claims (7 days apart), or one inpatient claim with at least one of the following diagnosis codes in any position using all available claims prior to the index date (including the index date):<br>1) ICD-9 codes: 427, 427.0, 427.1. 427.2, 427.3, 427.31, 427.32, 427.4, 427.41, 427.42, 427.5, 427.6, 427.60, 427.61, 427.69, 427.8, 427.81, 427.89, 427.9<br>2) ICD-10 codes: I47.0, I47.1, I47.2, I47.9, I48.0, I48.1, I48.2, I48.3, I48.4, I48.91, I48.92, I49.01, I49.02, I49.1, I49.2, I49.3, I49.40, I49.49, I49.5, I49.8, I49.9, R00.1
Chronic kidney disease[26,27]	Meeting at least one of the following criteria:<br>1) Either two outpatient claims (7 days apart), or one inpatient claim with at least one of the following diagnosis codes in any position using all available claims prior to the index date (including the index date):

© 2025 Derington CG et al. *JAMA Network Open*.

	a) ICD-9 codes: 016.0, 095.4, 189.0, 189.9, 223.0, 236.91, 250.4, 271.4, 274.1, 283.11, 403.x1, 403.x0, 404.x2, 404.x3, 404.x0, 404.x1, 440.1, 442.1, 447.3, 572.4, 580–588, 591, 642.1, 646.2, 753.12–753.17, 753.19, 753.2, 794.4<br>b) ICD-10 codes: A18.11, A52.75, C64.9, C68.9, D30.00, D41.00, D41.20, D59.3, E10.21, E10.29, E11.21, E11.29, E7.48, I120, I12.9, I13.0, I131.0, I131.1, I132, I70.1, I72.2, K76.7, M10.30, N0.03, N0.08, N0.09, N0.13, N0.22, N0.32, N0.33, N0.35, N0.38, N0.39, N0.40, N0.43, N0.44, N0.48, N0.49, N0.52, N0.55, N0.58, N0.59, N08, N13.30, N17.0, N17.1, N17.2, N17.8, N17.9, N18.1, N18.2, N18.3, N18.4, N18.5, N18.6, N18.9, N19, N25.0, N25.1, N25.81, N25.89, N25.9, N26.9,Q61.02, Q6119, Q61.2, Q61.3, Q61.4, Q61.5, Q61.8, Q621.0, Q621.1, Q621.2, Q62.31, Q62.39, R94.4.<br>2) Estimated glomerular filtration rate of <60 mL/min/1.73 m^2 on 2 separate visits at least 30 days or more apart in the one-year pre-index period. To account for data/reading errors, only eGFR values between 0 and 250 will be considered.<br>3) Meets criteria for end-stage kidney disease or dialysis, as below.
Chronic Liver Disease[28]	Either two outpatient claims (7 days apart), or one inpatient claim with at least one of the following diagnosis codes in any position using all available claims prior to the index date (including the index date):<br>1) ICD-9 codes: 070.22, 070.23, 070.32, 070.33, 070.44, 070.54, 456.0, 456.1, 456.20, 456.21, 570.x, 571.x, 572.x, 573.x, V42.7<br>2) ICD-10 codes: B18.0-B18.2, B25.1, I85.00, I85.01, I85.10, I85.11, K70.0, K70.10, K70.11, K70.2, K70.30, K70.31, K70.40, K70.41, K70.9, K71.0, K71.10, K71.11, K71.2, K71.3, K71.50, K71.51, K71.6, K71.7, K71.8, K71.9, K72.00, K72.01, K72.10, K72.11, K72.90, K72.91, K73.0, K73.1, K73.2, K73.8, K73.9, K74.0, K74.1, K74.2, K74.3, K74.4, K74.5, K74.60, K74.69, K75.0, K75.1m K75.2, K75.3, K75.4, K75.81, K75.89, K75.9, K76.0, K76.1, K76.2, K76.3, K76.4, K76.5, K76.6, K76.7, K76.81, K76.89, K76.9, K77.x, Z48.23, Z94.4
Cirrhosis [28]	Either two outpatient claims (7 days apart), or one inpatient claim with at least one of the following diagnosis codes in any position using all available claims prior to the index date (including the index date):<br>1) ICD-9 codes: 070.0, 070.2, 070.20, 070.21, 070.4, 070.41, 070.42, 070.43, 070.49, 070.6, 070.71, 571.2, 571.5, 571.6<br>2) ICD-10 codes: B15.0, B16.0, B16.2, B17.11, B19.0, B19.11, B19.21 K70.30, K70.31, K70.41, K71.11, K71.7, K72.01, K72.11, K72.91, K74.3, K74.4, K74.5, K74.60, K74.69, P78.81
Diabetes [28]	Meeting one of the two criteria below:

© 2025 Derington CG et al. *JAMA Network Open*.

	1) Either two outpatient claims (7 days apart), or one inpatient claim with at least one of the following diagnosis codes in any position using all available claims prior to the index date (including the index date):<br>a) ICD-9 codes: 250.xx, 357.2, 362.0x, or 366.41.<br>b) ICD-10 codes: E08.36, E08.42, E09.36, E09.42, E10.10, E10.11, E10.29, E10.311, E10.319, E10.36, E10.39, E10.40, E10.42, E10.51, E10.618, E10.620, E10.621, E10.622, E10.628, E10.630, E10.638, E10.641, E10.649, E10.65, E10.69, E10.8, E10.9, E11.00, E11.01, E11.29, E11.311, E11.319, E11.329, E11.339, E11.349, E11.359, E11.36, E11.39, E11.40, E11.42, E11.51, E11.618, E11.620, E11.621, E11.622, E11.628, E11.630, E11.638, E11.641, E11.649, E11.65, E11.69, E11.8, E11.9, E13.10, E13.36, E13.42.<br>2) ≥1 pharmacy claim for an oral antidiabetic drug fill or insulin in the one-year prior to the index date (including the index date).
Depression[28]	Either two outpatient claims (7 days apart), or one inpatient claim with at least one of the following diagnosis codes in any position using all available claims prior to the index date (including the index date):<br>1) ICD-9 codes: 296.2, 296.3, 296.5, 300.4, 309.x, or 311.<br>2) ICD-10 codes: F20.4, F31.3-F31.5, F32.x, F33.x, F34.1, F41.2, or F43.2.
Drug/substance use disorder	Either two outpatient claims (7 days apart), or one inpatient claim with at least one of the following diagnosis codes in any position using all available claims prior to the index date (including the index date):<br>1) ICD-9 codes: 292.0, 292.8x, 292.9, 304.0x, 304.1x, 304.2x, 304.3x, 304.4x, 304.5x, 304.6x, 304.7x, 304.8x, 304.9x, 305.2x, 305.3x, 305.4x, 305.5x, 305.6x, 305.7x, 305.8x, 305.9x, 648.3x<br>2) ICD-10 codes: F11.x, F12.x, F13.x, F14.x, F15.x, F16.x, F17.x, F18.x, F19.x, F55.x, O99.32x
End-stage kidney disease or dialysis[31]	Either two outpatient claims (7 days apart), or one inpatient claim with at least one of the following diagnosis codes in any position using all available claims prior to the index date (including the index date) with stop codes of 602, 603, 604, 605, 606, 607, 609, 610, or 611:<br>1) ICD-9 codes: 585.5.<br>2) ICD-10 codes: N18.6.
Frailty status	Defined using the 31-item VA Frailty Index (VA-FI) (see below). Based on the VA-FI, Veterans will be categorized into non-frail (FI ≤0.21) or frail (FI >0.21).

© 2025 Derington CG et al. *JAMA Network Open*.

VA Frailty Index (VA-FI)[32,36–38]	31-item index used to define frailty. Ratio of the sum of the number of health deficits to the total number of health factors evaluated. For example, a Veteran with 10 of 30 possible deficits would have an FI of 10/30 = 0.33. The 31 variables used to calculate the VA-FI have been validated in the Veterans Health Affairs system as described by Orkaby et al.[24]
Heart failure with reduced ejection fraction (systolic HF)	Meeting one of the three criteria below. Criteria below are sequentially applied.<br>1) PREFERENCE: Left ventricular ejection fraction (LVEF) value ≤40 in the **five-year** pre-index period (extended from one year due to infrequency of measurement in clinical practice). If there are multiple measurements on one date or if a range is provided, we will take the average of the two measurements.<br>2) If no LVEF measurement is available, then we will look for either two outpatient claims (7 days apart), or one inpatient claim with at least one of the following diagnosis codes in any position using all available claims prior to the index date (including the index date):<br>a) ICD-9 codes: 428.0x, 428.1x, 428.2x, or 428.4x.<br>b) ICD-10 codes: I50.1, I50.2x, I50.4x, or I50.9.<br>3) Finally, if a patient does not have an LVEF or one of the codes above, we will look for at least one prescription for sacubitril/valsartan in the 104 days (90 days + 14-day grace period) prior to the index date (including the index date).
Migraine	Meeting one of the following criteria:<br>1) Either two outpatient claims (7 days apart), or one inpatient claim with at least one of the following diagnosis codes in any position using all available claims prior to the index date (including the index date):<br>a) ICD-9 codes: 346.0, 346.1, 346.2, 346.3, 346.4, 346.5, 346.7, 346.8, 346.9<br>b) ICD-10 codes: G43.109A, G43.019, G43.B0, G43.419, G43.839, G43.519, G43.619, G43.719, G43.819, G43.919<br>2) ≥1 pharmacy dispenses for any of the following medications in the 104 days (90 days + 14-day grace period) prior to each Veteran's index date (including the index date): sumatriptan, naratriptan, zolmitriptan, rizatriptan, almotriptan, frovatriptan, eletriptan, butalbital, dihydroergotamine, ergotamine, erenumab, galcanezumab, fremanezumab, eptinezumab, rimegepant, atogepant, ubrogepant, lasmiditan.
Myocardial infarction[28,29]	Either two outpatient claims (7 days apart), or one inpatient claim with at least one of the following diagnosis codes in any position using all available claims prior to the index date (including the index date):<br>1) ICD-9 codes: 410.x

© 2025 Derington CG et al. *JAMA Network Open*.

	2) ICD-10 codes: I21.x, I22.x, or I25.2
Obstructive sleep apnea	Either two outpatient claims (7 days apart), or one inpatient claim with at least one of the following diagnosis codes in any position using all available claims prior to the index date (including the index date):<br>1) ICD-9 code: 327.2X, 327.23, 327.29, 780.51, 780.53, 780.57<br>2) ICD-10 code: G47.30, G47.33, G47.39
Peripheral Arterial Disease (PAD)[28,29]	Either two outpatient claims (7 days apart), or one inpatient claim with at least one of the following diagnosis codes in any position using all available claims prior to the index date (including the index date) Meeting one of the two criteria below:<br>1) ICD codes:<br>a) ICD-9 codes: 440.20-440.24, 440.31, 444.2, 443.9, or 444.81.<br>b) ICD-10 codes: I70.209, I70.219, I70.229, I70.25, I70.269, I70.499, I73.9.<br>c) ICD-9 procedure codes: 38.x, 39.22, 39.24, 29.36, 39.26, 39.28, 39.5, or 39.50.<br>2) CPT codes: 37205 or 75962.
Prior coronary revascularization	Meeting BOTH criteria 1 and 2 below.<br>1) Defined by ≥1 inpatient or outpatient procedure with one of the following using all available claims prior to the index date (including the index date):<br>a) CPT code for coronary revascularization: 33510-33519, 33521-33523, 33530, 33533-33536, 92920, 92921, 92924, 92925, 92928, 92929, 92933, 92934, 92937, 92938, 92941, 92943, 92944, 92980, 92981, 92982, 92984, or 92996<br>b) ICD-9 procedure code (any position) of 00.66, 36.0, 36.10-36.19, or 36.2<br>c) ICD-10 procedure code starting with any of the following: 0210, 0211, 0212, 0213, 0270, 0271, 0272, 0273, 02C0, 02C1, 02C2, 02C3, or 3E07.<br>2) Meet at least one of the following criteria:<br>a) Have no inpatient claims with a discharge diagnosis code for acute myocardial infarction (ICD-9 codes 410.x0 or 410.x1 or ICD-10 codes I21.xx or I22.xx) within 60 days prior to the procedure.<br>b) Have primary discharge diagnosis codes for non-elective CHD-related hospitalization prior to the index date (including the index date):<br>a. Arrhythmia: ICD-9 diagnosis code of 427.xx [except 427.5] or ICD-10 diagnosis code of I47.1, I47.2, I47.9, I48.91, I48.92, I49.01, I49.02, I49.1, I49.3, I49.40, I49.49, I49.5, I49.8, I49.9, R00.1.

© 2025 Derington CG et al. *JAMA Network Open*.

	b. Cardiac arrest: ICD-9 diagnosis code of 427.5, or ICD-10 diagnosis code of I46.9.<br>c. Heart failure: ICD-9 diagnosis code of 402.01, 402.11, 402.91, 404.01, 404.03, 404.11, 404.13, 404.91, 404.93, or 428.x, or ICD-10 diagnosis code of I11.0, I13.0, I13.2, I50.1, I50.20, I50.21, I50.22, I50.23, I50.30, I50.31, I50.32, I50.33, I50.40, I50.41, I50.42, I50.43, or I50.9.<br>d. Unstable angina: ICD-9 diagnosis code of 411.xx or ICD-10 diagnosis code of I20.0, I24.0, I24.1, I24.8.
Any Stroke[34]	Meeting one of the two criteria below using all available claims prior to the index date (including the index date):<br>1) ICD-9 codes:<br>a) ≥1 inpatient claim with a discharge diagnosis code (primary or secondary position) of 431, 432.X, 433.x1, 434.xx, 433.x0, 436, 437.x, 438.xx.<br>b) ≥1 outpatient claim with a diagnosis code (any position) of 431, 432.X, 433.x1, 434.xx, 433.x0, 436, 437.x, 438.xx.<br>2) ICD-10 codes:<br>a) ≥1 inpatient discharge diagnosis code (primary or secondary position) of I60.xx, I61.x, I62.xx, I63.xxx, I66.xx, I65.xx, I67.xxx, I68.x, I69.xxx<br>b) ≥1 outpatient claim with diagnosis code (any position) of I60.xx, I61.x, I62.xx, I63.xxx, I66.xx, I65.xx, I67.xxx, I68.x, I69.xxx<br>c) ≥1 inpatient ICD-10 procedure code of 03CH0ZZ, 03CH4ZZ, 03CJ0ZZ, 03CJ4ZZ, 03CK0ZZ, 03CK4ZZ, 03CL0ZZ, 03CL4ZZ, 03CM0ZZ, 03CM4ZZ, 03CN0ZZ, 03CN4ZZ, 03RH07Z, 03RH0JZ, 03RH0KZ, 03RH47Z, 03RH4JZ, 03RH4KZ, 03RJ07Z, 03RJ0JZ, 03RJ0KZ, 03RJ47Z, 03RJ4JZ, 03RJ4KZ,03RK07Z, 03RK0JZ, 03RK0KZ, 03RK47Z, 03RK4JZ, 03RK4KZ, 03RL07Z, 03RL0JZ, 03RL0KZ, 03RL47Z, 03RL4JZ, 03RL4KZ, 03RM07Z, 03RM0JZ, 03RM0KZ, 03RM47Z, 03RM4JZ, 03RM4KZ, 03RN07Z, 03RN0JZ, 03RN0KZ, 03RN47Z, 03RN4JZ, or 03RN4KZ.<br>3) CPT codes: ≥1 inpatient or outpatient claim with a CPT code for carotid revascularization of 35301, 35390, 37215, 37216, 0005T, 0075T, or 0076.
Concomitant Medication Use	
Statin use	Defined as one or more outpatient pharmacy dispenses for a statin medication in the 104 days (90 days + 14-day grace period) prior to each Veteran's index date (including the index date). Statin medications include: atorvastatin, rosuvastatin, simvastatin, fluvastatin, pitavastatin, pravastatin, or lovastatin.

© 2025 Derington CG et al. *JAMA Network Open*.

Aspirin use	Defined as one or more outpatient pharmacy dispenses for aspirin in the 104 days (90 days + 14-day grace period) prior to each Veteran's index date (including the index date).
ACEI use	Defined as a pharmacy dispense for one of the following oral medications: benazepril, captopril, enalapril, fosinopril, lisinopril, moexipril, perindopril, quinapril, ramipril, trandolapril, spirapril.
ARB use	Defined as a pharmacy dispense for one of the following oral medications: azilsartan, candesartan, eprosartan, losartan, olmesartan, telmisartan, valsartan.
β-blocker	Defined as a pharmacy dispense for one of the following oral medications: acebutolol, nebivolol, atenolol, betaxolol, bisoprolol, metoprolol, metoprolol, pindolol, penbutolol, carvedilol, labetalol, nadolol, propranolol, timolol
Calcium channel blocker	Defined as a pharmacy dispense for one of the following oral medications: amlodipine, felodipine, isradipine, nicardipine, nifedipine, nisoldipine, diltiazem, verapamil
Other antihypertensive use.	Defined as a pharmacy dispense for an alpha-1 blocker (doxazosin, prazosin, terazosin), centrally-acting agent (clonidine (oral or patch), guanabenz, guanadrel, guanethidine, guanfacine, deserpidine, methyldopa, reserpine), vasodilator (hydralazine or reserpine), direct renin inhibitor (aliskiren), aldosterone receptor antagonist (spironolactone, eplerenone), loop diuretic (bumetanide, ethacrynic acid, furosemide, torsemide), or potassium-sparing diuretic (amiloride, triamterene).
Thiazide or thiazide-like diuretic	Defined as a pharmacy dispense for one of the following oral medications: Bendroflumethiazide, chlorothiazide, chlorthalidone, hydrochlorothiazide, methyclothiazide, polythiazide, hydroflumethiazide, trichlormethiazide, quinethazone, indapamide, metolazone
Number of antihypertensive medications	Sum of the number of antihypertensive medications dispensed in the pre-index period with a days' supply that overlaps with the Veteran's index date (including the index date).
Healthcare utilization	
Number of primary care visits	Defined as the continuous number of outpatient primary care encounters (160, 170, 172, 176, 210, 301, 309, 318, 322, 323, 342, 348, 350, 348, 394) in the one-year pre-index period (including the index date).
Number of hospitalizations	Defined as the continuous number of all-cause hospitalizations in the one-year pre-index period (including the index date).
Number of emergency department visits	Defined as the continuous number of emergency department visits in the one-year pre-index period (including the index date).
Academic setting[31]	Defined by the patient's sta3n variable; coded as dichotomous (yes/no) based on http://www.friendsofva.org/resources/2012/finalvainfrastructurereport.pdf.
Urban setting [31]	Defined by the patient's sta3n variable; coded as rural vs. non-rural based on https://www.ruralhealth.va.gov/docs/atlas/CHAPTER_02_RHRI_Pts_treated_at_VAMCs.pdf. Facilities with red lettering in Table 2 are coded as rural.

© 2025 Derington CG et al. *JAMA Network Open*.

eTable 2. Missingness Table, Overall and by Compelling Indication Status

	**Overall**	**Compelling indication for a β-blocker**	
Characteristic		Yes	No
Index year category	0	0	0
Age (years), mean(SD)	0	0	0
Male sex, n (%)	0	0	0
Female sex, n (%)	0	0	0
Race/Ethnicity, n(%)	0	0	0
county-level per-capita income (US Dollars), mean (SD)	631,123 (20.11%)	39,354 (17.61%)	591,769 (20.30%)
Priority group status, n(%)	0	0	0
Current Smoker, n (%)	0	0	0
Homeless or history of homelessness, n (%)	0	0	0
Body mass index, (kg/m^2) mean (SD)	570,197 (18.17%)	37,699 (16.87%)	532,498 (18.27%)
Systolic blood pressure category (mm Hg), n (%)	322,711 (10.28%)	26,115 (11.69%)	296,596 (10.18%)
Diastolic blood pressure category (mm Hg), n (%)	322,711 (10.28%)	26,115 (11.69%)	296,596 (10.18%)
Total cholesterol (mg/dL), median [IQR]	949,492 (30.25%)	52,219 (23.37%)	897,273 (30.78%)
High-density lipoprotein cholesterol (mg/dL), median [IQR]	1,017,226 (32.41%)	56,321 (25.20%)	960,905 (32.97%)
Low-density lipoprotein cholesterol (mg/dL), median [IQR]	1,099,879 (35.05%)	63,648 (28.48%)	1,036,231 (35.55%)
Triglycerides (mg/dL),median [IQR]	1,053,805 (33.58%)	57,636 (25.79%)	996,169 (34.18%)
Hemoglobin A1c (%), median [IQR]	1,789,899 (57.03%)	107,355 (48.04%)	1,682,544 (57.72%)
Estimated glomerular filtration rate (mL,min/1.73m^2), mean (SD)	696,188 (22.18%)	28,377 (12.70%)	667,811 (22.91%)
BL_HeartRate	95,265 (3.04%)	3,971 (1.78%)	91,294 (3.13%)
Alcohol abuse	0	0	0
Aortic aneurysm/disease	0	0	0
Angina	0	0	0
Atrial fibrillation or arrhytmia	0	0	0
Chronic kidney disease	0	0	0
Chronic liver disease or cirrhosis	0	0	0
Diabetes	0	0	0
Depression	0	0	0
Drug/substance use	0	0	0
End-stage kidney disease or dialysis	0	0	0
Frail based on the VA Frailty index	0	0	0
Heart Failure	0	0	0
Migraine	0	0	0
Myocardial infarction	0	0	0
Obstructive sleep apnea	0	0	0
Peripherial artery disease	0	0	0
Prior coronary revascularization	0	0	0
Stroke	0	0	0
Aspirin, n (%)	0	0	0
Statin, n (%)	0	0	0

© 2025 Derington CG et al. *JAMA Network Open*.

	**Overall**	**Compelling indication for a β-blocker**	
Characteristic		Yes	No
Primary care visits	0	0	0
Hospitalizations	0	0	0
Emergency department visits	0	0	0
Veterans Integrated Service Network Region	0	0	0
Academic setting	0	0	0
Urban setting	0	0	0

© 2025 Derington CG et al. *JAMA Network Open*.

eTable 3. Characteristics of Those Missing vs Not Missing ≥1 Key Variables in the Fully Adjusted Analysis, Overall and by Compelling Indication Status

	Overall		Compelling indication for a β-blocker			
			Yes		No	
	Missing	Not missing	Missing	Not missing	Missing	Not missing
Variable	N = 2,411,548	N = 726,756	N = 161,751	N = 61,751	N = 2,249,797	N = 665,025
Index year category						
2000-2005	838,103 (34.8%)	122,128 (16.8%)	48,086 (29.7%)	8,728 (14.1%)	790,017 (35.1%)	113,400 (17.1%)
2006-2011	686,405 (28.5%)	228,147 (31.4%)	42,462 (26.3%)	17,584 (28.5%)	643,943 (28.6%)	210,563 (31.7%)
2012-2017	473,101 (19.6%)	248,692 (34.2%)	32,362 (20.0%)	21,316 (34.5%)	440,739 (19.6%)	227,376 (34.2%)
2018-2022	413,939 (17.2%)	127,789 (17.6%)	38,841 (24.0%)	14,103 (22.8%)	375,098 (16.7%)	113,686 (17.1%)
Demographics						
Age (years), mean (SD)	61 (13.3)	61 (12.0)	65 (12.3)	65 (11.1)	61 (13.3)	61 (12.0)
Male sex, n (%)	2,273,171 (94.3%)	685,317 (94.3%)	155,874 (96.4%)	59,489 (96.4%)	2,117,297 (94.1%)	625,828 (94.1%)
Race/Ethnicity, n (%)						
Non-Hispanic White	1,547,924 (64.2%)	480,203 (66.1%)	108,594 (67.1%)	43,246 (70.1%)	1,439,330 (64.0%)	436,957 (65.7%)
Non-Hispanic Black	370,404 (15.4%)	120,232 (16.5%)	21,019 (13.0%)	8,415 (13.6%)	349,385 (15.5%)	111,817 (16.8%)
Hispanic	107,720 (4.5%)	42,087 (5.8%)	6,234 (3.9%)	3,033 (4.9%)	101,486 (4.5%)	39,054 (5.9%)
Other*	50,848 (2.1%)	15,950 (2.2%)	2,868 (1.8%)	1,142 (1.8%)	47,980 (2.1%)	14,808 (2.2%)
Unknown/Missing	334,652 (13.9%)	68,284 (9.4%)	23,036 (14.2%)	5,895 (9.5%)	311,616 (13.9%)	62,389 (9.4%)
Social and Behavioral						
Priority group status, n (%)						
1 (highest need)	369,393 (15.3%)	107,667 (14.8%)	27,668 (17.1%)	10,102 (16.4%)	341,725 (15.2%)	97,565 (14.7%)
2 through 8	1,572,386 (65.2%)	500,648 (68.9%)	111,206 (68.8%)	44,708 (72.4%)	1,461,180 (64.9%)	455,940 (68.6%)
Unknown/missing	469,769 (19.5%)	118,441 (16.3%)	22,877 (14.1%)	6,921 (11.2%)	446,892 (19.9%)	111,520 (16.8%)
Current smoker	382,324 (15.9%)	134,137 (18.5%)	36,906 (22.8%)	15,634 (25.3%)	345,418 (15.4%)	118,503 (17.8%)

© 2025 Derington CG et al. *JAMA Network Open*.

Variable	Overall		Compelling indication for a β-blocker			
			Yes		No	
	Missing	Not missing	Missing	Not missing	Missing	Not missing
	N = 2,411,548	N = 726,756	N = 161,751	N = 61,751	N = 2,249,797	N = 665,025
Homeless or history of homelessness, n (%)	63,364 (2.6%)	25,750 (3.5%)	6,194 (3.8%)	3,146 (5.1%)	57,170 (2.5%)	22,604 (3.4%)
Clinical/Laboratory Measurements						
Medical Conditions, n (%)						
Alcohol use disorder	74,722 (3.1%)	25,980 (3.6%)	12,222 (7.6%)	4,599 (7.5%)	62,500 (2.8%)	21,381 (3.2%)
Aortic aneurysm/disease	6,694 (0.3%)	3,036 (0.4%)	6,694 (4.1%)	3,036 (4.9%)	0 (0.0%)	0 (0.0%)
Angina	7,185 (0.3%)	3,018 (0.4%)	7,185 (4.4%)	3,018 (4.9%)	0 (0.0%)	0 (0.0%)
Atrial fibrillation or arrhythmia	45,538 (1.9%)	19,087 (2.6%)	45,538 (28.2%)	19,087 (30.9%)	0 (0.0%)	0 (0.0%)
Chronic kidney disease	40,000 (1.7%)	21,939 (3.0%)	11,885 (7.3%)	5,893 (9.5%)	28,115 (1.2%)	16,046 (2.4%)
Chronic liver disease or cirrhosis	23,982 (1.0%)	10,895 (1.5%)	23,982 (14.8%)	10,895 (17.6%)	0 (0.0%)	0 (0.0%)
Diabetes	161,184 (6.7%)	171,791 (23.6%)	22,991 (14.2%)	20,896 (33.9%)	138,193 (6.1%)	150,895 (22.7%)
Depression	191,009 (7.9%)	82,493 (11.4%)	18,798 (11.6%)	9,546 (15.5%)	172,211 (7.7%)	72,947 (11.0%)
Drug/substance use	52,085 (2.2%)	19,724 (2.7%)	10,038 (6.2%)	4,412 (7.1%)	42,047 (1.9%)	15,312 (2.3%)
End-stage kidney disease or dialysis	544 (0.0%)	187 (0.0%)	266 (0.2%)	88 (0.1%)	278 (0.0%)	99 (0.0%)
Frail based on the VA Frailty index†	42,862 (1.8%)	24,516 (3.4%)	14,247 (8.8%)	8,311 (13.5%)	28,615 (1.3%)	16,205 (2.4%)
Heart failure with reduced ejection fraction	90,022 (3.7%)	30,810 (4.2%)	90,022 (55.7%)	30,810 (49.9%)	0 (0.0%)	0 (0.0%)
Myocardial infarction	7,210 (0.3%)	2,769 (0.4%)	7,210 (4.5%)	2,769 (4.5%)	0 (0.0%)	0 (0.0%)
Obstructive sleep apnea	67,802 (2.8%)	31,593 (4.3%)	9,793 (6.1%)	5,089 (8.2%)	58,009 (2.6%)	26,504 (4.0%)
Peripheral artery disease	19,625 (0.8%)	9,321 (1.3%)	5,678 (3.5%)	2,632 (4.3%)	13,947 (0.6%)	6,689 (1.0%)
Prior coronary revascularization	2,244 (0.1%)	1,514 (0.2%)	2,244 (1.4%)	1,514 (2.5%)	0 (0.0%)	0 (0.0%)
Stroke	83,094 (3.4%)	28,165 (3.9%)	10,849 (6.7%)	4,886 (7.9%)	72,245 (3.2%)	23,279 (3.5%)

© 2025 Derington CG et al. *JAMA Network Open*.

Variable	Overall		Compelling indication for a β-blocker			
			Yes		No	
	Missing	Not missing	Missing	Not missing	Missing	Not missing
	N = 2,411,548	N = 726,756	N = 161,751	N = 61,751	N = 2,249,797	N = 665,025
Medication Use						
Aspirin, n (%)	228,233 (9.5%)	80,205 (11.0%)	31,461 (19.5%)	12,155 (19.7%)	196,772 (8.7%)	68,050 (10.2%)
Statin, n (%)	758,480 (31.5%)	296,134 (40.7%)	61,887 (38.3%)	27,545 (44.6%)	696,593 (31.0%)	268,589 (40.4%)
Healthcare Utilization in Prior Year						
Primary care visits, median [IQR]	2 (1.0, 4)	3 (2.0, 6)	4 (2.0, 7)	5 (3.0, 9)	2 (1.0, 4)	3 (2.0, 5)
Had ≥1 hospitalization, n (%)	140,714 (5.8%)	50,247 (6.9%)	44,532 (27.5%)	17,574 (28.5%)	96,182 (4.3%)	32,673 (4.9%)
Had ≥1 emergency department visit, n (%)	357,523 (14.8%)	115,503 (15.9%)	45,856 (28.3%)	19,369 (31.4%)	311,667 (13.9%)	96,134 (14.5%)
Facility characteristics						
Veterans Integrated Service Network Region#						
Northeast	751,018 (31.1%)	203,470 (28.0%)	57,374 (35.5%)	20,401 (33.0%)	693,644 (30.8%)	183,069 (27.5%)
Southeast	776,992 (32.2%)	251,121 (34.6%)	47,738 (29.5%)	20,306 (32.9%)	729,254 (32.4%)	230,815 (34.7%)
Continental	511,115 (21.2%)	142,011 (19.5%)	33,341 (20.6%)	10,857 (17.6%)	477,774 (21.2%)	131,154 (19.7%)
Pacific	372,423 (15.4%)	130,154 (17.9%)	23,298 (14.4%)	10,167 (16.5%)	349,125 (15.5%)	119,987 (18.0%)
Academic setting	1,591,226 (66.0%)	498,891 (68.6%)	114,751 (70.9%)	44,360 (71.9%)	1,476,475 (65.6%)	454,531 (68.3%)
Urban setting	135,581 (5.6%)	33,479 (4.6%)	7,459 (4.6%)	2,582 (4.2%)	128,122 (5.7%)	30,897 (4.6%)

Those who are missing at least one of the following are classified as “Missing ≥1 variable”: county-level per-capita income, body mass index, systolid blood pressure, diastolic blood pressure, total cholesterol, high-density lipoprotein cholesterol, low-density lipoprotein cholesterol, triglycerides, hemoglobin A1c, estimated glomerular filtration rate, and heart rate.

*Includes Asian American, American Indian or Alaskan Native, and Native Hawaiian or Other Pacific Islander.
ΨAccording to the US Bureau of Economic Analysis, matched to ZIP or FIPS code (https://apps.bea.gov/regional/histdata/index.cfm)

© 2025 Derington CG et al. *JAMA Network Open*.

† Ratio of the sum of the number of health deficits relative to 31 health factors evaluated, ranging from 0 to 1. 0 indicates no frailty, while non-zero numbers indicate some degree of frailty. Non-frail was defined as a calculated frailty index ≤0.21, and frail was defined as a calculated frailty index >0.21.
‡ Defined as: aldosterone receptor antagonist, centrally-acting drug, direct arterial vasodilator, direct renin inhibitor, loop diuretic, and potassium sparing diuretic
# Categorized according to the VA regional offices map (https://www.benefits.va.gov/benefits/offices.asp). The Northeast region was comprised of VISNs 1, 2, 4, 5, 10, and 12. The Southeast region was comprised of VISNs 6, 7, 8, 9, and 16. The Continental region consisted of VISNs 15, 17, 18, 19, and 23. Finally, the Pacific region was comprised of VISNs 20, 21, and 22.

ED: emergency department; VA: Veterans Affairs

© 2025 Derington CG et al. *JAMA Network Open*.

eTable 4. Initiation of Specific β-Blocker Regimens

N initiating a β-blocker	Overall<br>N = 774,821	Compelling indication Yes<br>N = 90,776	Compelling indication No<br>N = 684,045
Initiated β-blocker monotherapy, n (%)	372,330 (48.1)	39,852 (43.9)	332,478 (48.6)
Initiated β-blocker with ≥1 first-line medication (ACEI, ARB, CCB, or thiazide), with or without “other” agents, n (%)	373,882 (48.3)	43,799 (48.2)	330,083 (48.3)
Initiated β-blocker with ≥1 “other” agent only (no first-line medications initiated), n (%)	28,609 (3.7)	7,125 (7.8)	21,484 (3.1)

Other = aldosterone receptor antagonist, centrally-acting drug, direct arterial vasodilator, direct renin inhibitor, loop diuretic, and potassium sparing diuretic

© 2025 Derington CG et al. *JAMA Network Open*.

eTable 5. Prevalence Ratios and 95% CIs for Factors Associated With Initiation of β-Blockers Among Those Without Compelling Indications

Variable	Unadjusted (Model 1)	Age- and sex- adjusted (Model 2)	Fully adjusted (Model 3)
Index year category			
2000-2005	1 (Ref)	1 (Ref)	1 (Ref)
2006-2011	0.86 (0.85,0.86)	0.91 (0.91,0.92)	0.99 (0.98,1.01)
2012-2017	0.80 (0.80,0.81)	0.86 (0.86,0.87)	1.02 (1.01,1.04)
2018-2022	0.70 (0.70,0.70)	0.74 (0.74,0.75)	0.91 (0.90,0.93)
Demographics			
Age, per 10 years	1.16 (1.16,1.16)	NA	1.05 (1.04,1.05)
Female sex vs. male sex	0.83 (0.82,0.84)	NA	1.11 (1.09,1.14)
Race/Ethnicity, n (%)			
Non-Hispanic White	1 (Ref)	1 (Ref)	1 (Ref)
Non-Hispanic Black	0.62 (0.61,0.62)	0.68 (0.68,0.69)	0.74 (0.73,0.76)
Hispanic	0.67 (0.67,0.68)	0.72 (0.71,0.73)	0.75 (0.73,0.77)
Other*	0.79 (0.77,0.80)	0.84 (0.82,0.85)	0.89 (0.86,0.92)
Unknown/Missing	1.02 (1.01,1.02)	0.98 (0.97,0.98)	0.96 (0.94,0.97)
Social and Behavioral			
County-level per-capita income (US Dollars)ψ, per $10,000 USD	1.00 (1.00,1.00)	1.00 (1.00,1.00)	1.00 (1.00,1.00)
Priority group status			
1	1 (Ref)	1 (Ref)	1 (Ref)
2 through 8	1.15 (1.14,1.15)	1.05 (1.04,1.05)	1.01 (1.00,1.02)
Unknown/missing	1.33 (1.32,1.34)	1.27 (1.26,1.27)	1.15 (1.14,1.17)
Current smoker vs. former or never	0.93 (0.92,0.93)	0.99 (0.99,1.00)	1.04 (1.02,1.05)
Homeless or history of homelessness	0.79 (0.78,0.80)	0.89 (0.88,0.90)	0.97 (0.94,0.99)
Clinical/Laboratory Measurements			
Body mass index (kg/m^2), per 5 kg/m^2	0.99 (0.98,0.99)	1.03 (1.03,1.03)	1.03 (1.02,1.03)
SBP, per 10 mm Hg	0.92 (0.92,0.92)	0.92 (0.92,0.92)	0.94 (0.94,0.95)
DBP, per 10 mm Hg	0.83 (0.83,0.83)	0.86 (0.86,0.87)	0.95 (0.95,0.96)
Heart rate, per 10 bpm	0.88 (0.88,0.89)	0.91 (0.91,0.91)	0.92 (0.92,0.92)
Total cholesterol (mg/dL), per 10 mg/dL	0.96 (0.96,0.96)	0.97 (0.97,0.97)	0.99 (0.99,1.00)
High-density lipoprotein cholesterol (mg/dL), per 10 mg/dL	0.92 (0.91,0.92)	0.91 (0.91,0.91)	0.96 (0.95,0.96)
Low-density lipoprotein cholesterol (mg/dL), per 10 mg/dL	0.95 (0.95,0.95)	0.96 (0.96,0.96)	0.98 (0.97,0.98)
Triglycerides (mg/dL), per 10 mg/dL	1.00 (1.00,1.00)	1.01 (1.01,1.01)	1.00 (1.00,1.01)
Hemoglobin A1c (%), per 0.5%	1.00 (1.00,1.00)	1.00 (1.00,1.00)	1.00 (1.00,1.00)
Estimated glomerular filtration rate (mL/min/1.73m^2), per 10 mL/min/1.73m^2	0.92 (0.92,0.92)	0.95 (0.95,0.95)	0.95 (0.95,0.95)
Medical Conditions			

© 2025 Derington CG et al. *JAMA Network Open*.

Alcohol use disorder	0.88 (0.87,0.90)	1.00 (0.99,1.01)	1.13 (1.10,1.17)
Chronic kidney disease	1.09 (1.07,1.11)	1.01 (0.99,1.03)	0.98 (0.96,1.01)
Diabetes	0.76 (0.76,0.77)	0.74 (0.74,0.75)	0.68 (0.67,0.69)
Depression	0.79 (0.78,0.79)	0.88 (0.87,0.88)	0.91 (0.89,0.93)
Drug/substance use	0.75 (0.73,0.76)	0.85 (0.83,0.86)	0.87 (0.84,0.91)
End-stage kidney disease or dialysis	2.24 (2.03,2.46)	2.20 (1.99,2.43)	1.69 (1.39,2.06)
Frail based on the VA Frailty index†	1.43 (1.41,1.45)	1.25 (1.24,1.27)	1.28 (1.25,1.31)
Obstructive sleep apnea	0.68 (0.67,0.69)	0.75 (0.74,0.76)	0.87 (0.85,0.90)
Peripheral artery disease	1.25 (1.22,1.28)	1.16 (1.14,1.19)	1.12 (1.07,1.16)
Stroke	1.43 (1.41,1.44)	1.29 (1.28,1.30)	1.15 (1.13,1.18)
Medication Use			
Aspirin	1.74 (1.73,1.75)	1.67 (1.66,1.68)	1.49 (1.48,1.51)
Statin	1.56 (1.55,1.56)	1.47 (1.47,1.48)	1.32 (1.31,1.34)
Healthcare Utilization in Prior Year, median [IQR]			
Primary care visits, per 1 visit	0.97 (0.97,0.97)	0.97 (0.97,0.98)	0.99 (0.99,0.99)
Hospitalizations, per 1 hospitalization	1.09 (1.09,1.10)	1.12 (1.12,1.13)	1.15 (1.13,1.16)
Emergency department visits, per 1 visit	0.93 (0.93,0.94)	0.97 (0.96,0.97)	0.98 (0.98,0.99)
Facility characteristics			
Veterans Integrated Service Network Region#			
Northeast	1 (Ref)	1 (Ref)	1 (Ref)
Southeast	0.82 (0.82,0.83)	0.85 (0.85,0.86)	0.91 (0.90,0.92)
Continental	0.91 (0.90,0.91)	0.93 (0.92,0.93)	0.95 (0.94,0.96)
Pacific	0.87 (0.87,0.88)	0.89 (0.89,0.90)	0.93 (0.91,0.94)
Academic vs. non-academic setting	0.98 (0.98,0.99)	0.99 (0.98,0.99)	1.00 (0.99,1.01)
Urban vs. rural setting	1.07 (1.06,1.08)	1.06 (1.05,1.07)	1.00 (0.97,1.02)

*Includes Asian American, American Indian or Alaskan Native, and Native Hawaiian or Other Pacific Islander.

[ψ]According to the US Bureau of Economic Analysis, matched to ZIP or FIPS code (https://apps.bea.gov/regional/histdata/index.cfm)

[†] Ratio of the sum of the number of health deficits relative to 31 health factors evaluated, ranging from 0 to 1. 0 indicates no frailty, while non-zero numbers indicate some degree of frailty. Non-frail was defined as a calculated frailty index ≤0.21, and frail was defined as a calculated frailty index >0.21.

[#] Categorized according to the VA regional offices map (https://www.benefits.va.gov/benefits/offices.asp). The Northeast region was comprised of VISNs 1, 2, 4, 5, 10, and 12. The Southeast region was comprised of VISNs 6, 7, 8, 9, and 16. The Continental region consisted of VISNs 15, 17, 18, 19, and 23. Finally, the Pacific region was comprised of VISNs 20, 21, and 22.

The pseudo-R^2 values for Models 1 and 2 had median values of 0.00041 and 0.00773, respectively, while Model 3 had a pseudo-R^2 of 0.04545. The corresponding median deviance statistics were 1,980,483 for Model 1, 1,957,133 for Model 2, and 404,847 for Model 3.

The pseudo-R^2 and deviance statistics suggest improved model fit with increasing adjustment. Model 3, which includes all exposures jointly, had substantially higher pseudo-R^2 and lower deviance compared to Models 1 and 2, indicating better overall explanatory power. However, comparisons across models should be interpreted cautiously given differences in model structure and the number of models summarized.

© 2025 Derington CG et al. *JAMA Network Open*.

eTable 6. Initiation of β-Blocker Regimens and Specific β-Blockers Initiated Among Veterans With Incident Hypertension, 2000-2022, Including Migraine as a Compelling Indication

Population and medication regimen	Overall	Index year			
		2000-2005	2006-2011	2012-2017	2018-2022
With compelling indications	***N = 259,358***	***N = 63,293***	***N = 68,734***	***N = 63,863***	***N = 63,468***
Non-β-blocker regimen initiated	158,455 (61.1%)	39,275 (62.1%)	41,969 (61.1%)	37,421 (58.6%)	39,790 (62.7%)
β-blocker regimen initiated	100,903 (38.9%)	24,018 (37.9%)	26,765 (38.9%)	26,442 (41.4%)	23,678 (37.3%)
Atenolol	15,097 (15.0%)	7,596 (31.6%)	4,198 (15.7%)	2,224 (8.4%)	1,079 (4.6%)
Carvedilol	19,772 (19.6%)	2,597 (10.8%)	5,027 (18.8%)	6,729 (25.4%)	5,419 (22.9%)
Metoprolol	57,862 (57.3%)	12,477 (51.9%)	15,619 (58.4%)	15,125 (57.2%)	14,641 (61.8%)
Propranolol	7,187 (7.1%)	1,159 (4.8%)	1,701 (6.4%)	2,093 (7.9%)	2,234 (9.4%)
Other β-blocker	1,096 (1.1%)	219 (0.9%)	257 (1.0%)	294 (1.1%)	326 (1.4%)
Without compelling indications	***N = 2,878,946***	***N = 896,938***	***N = 845,818***	***N = 657,930***	***N = 478,260***
Non-β-blocker regimen initiated	2,205,028 (76.6%)	653,336 (72.8%)	649,341 (76.8%)	514,565 (78.2%)	387,786 (81.1%)
β-blocker regimen initiated	673,918 (23.4%)	243,602 (27.2%)	196,477 (23.2%)	143,365 (21.8%)	90,474 (18.9%)
Atenolol	232,103 (34.4%)	122,427 (50.3%)	68,010 (34.6%)	31,007 (21.6%)	10,659 (11.8%)
Carvedilol	56,862 (8.4%)	5,817 (2.4%)	13,461 (6.9%)	21,889 (15.3%)	15,695 (17.3%)
Metoprolol	345,719 (51.3%)	105,064 (43.1%)	106,295 (54.1%)	80,294 (56.0%)	54,066 (59.8%)
Propranolol	31,388 (4.7%)	7,917 (3.2%)	6,990 (3.6%)	8,385 (5.8%)	8,096 (8.9%)
Other β-blocker	8,372 (1.2%)	2,559 (1.1%)	1,882 (1.0%)	1,908 (1.3%)	2,023 (2.2%)

Cells are n (column proportion expressed as percentages), with the exception of the specific β-blocker rows, which are n (proportion of total initiating β-blocker, expressed as a percentage).

© 2025 Derington CG et al. *JAMA Network Open*.

eTable 7. Sensitivity Analysis Evaluating Factors Associated With Initiation of β-Blockers for Veterans Starting Antihypertensive Medications January 1, 2000, Through December 31, 2014, and January 1, 2015, Through December 31, 2022

	**Unadjusted (Model 1)**		**Age- and sex- adjusted (Model 2)**		**Fully adjusted (Model 3)**	
Variable	**2000-2014 N = 2,133,514**	**2015-2022 N = 801,308**	**2000-2014 N = 2,133,514**	**2015-2022 N = 801,308**	**2000-2014 N = 2,133,514**	**2015-2022 N = 801,308**
Index year, per each year	0.98 (0.98,0.98)	0.98 (0.97,0.98)	0.99 (0.99,0.99)	0.97 (0.97,0.97)	1.01 (1.00,1.01)	0.98 (0.97,0.98)
Demographics						
Age, per 10 years	0.85 (0.84,0.86)	0.85 (0.83,0.86)	NA	NA	1.08 (1.05,1.11)	1.16 (1.12,1.20)
Female sex vs. male sex	1.13 (1.13,1.14)	1.23 (1.22,1.23)	NA	NA	1.03 (1.02,1.03)	1.09 (1.08,1.10)
Race/Ethnicity, n (%)						
Non-Hispanic White	1 (Ref)	1 (Ref)	1 (Ref)	1 (Ref)	1 (Ref)	1 (Ref)
Non-Hispanic Black	0.62 (0.62,0.63)	0.63 (0.62,0.64)	0.68 (0.67,0.69)	0.71 (0.70,0.72)	0.75 (0.74,0.77)	0.73 (0.71,0.75)
Hispanic	0.69 (0.68,0.70)	0.67 (0.66,0.69)	0.72 (0.71,0.73)	0.76 (0.74,0.78)	0.76 (0.74,0.78)	0.75 (0.72,0.78)
Other*	0.81 (0.79,0.82)	0.78 (0.76,0.81)	0.85 (0.83,0.86)	0.87 (0.84,0.89)	0.89 (0.85,0.93)	0.90 (0.85,0.95)
Unknown/Missing	1.02 (1.01,1.02)	0.92 (0.90,0.93)	0.98 (0.97,0.98)	0.95 (0.93,0.96)	0.97 (0.96,0.99)	0.94 (0.91,0.97)
Social and Behavioral						
County-level per-capita income (US Dollars)ψ, per $10,000 USD	1.00 (1.00,1.00)	1.00 (1.00,1.00)	1.00 (1.00,1.00)	1.00 (1.00,1.00)	1.00 (1.00,1.00)	1.00 (1.00,1.00)
Priority group status						
1	1 (Ref)	1 (Ref)	1 (Ref)	1 (Ref)	1 (Ref)	1 (Ref)
2 through 8	1.09 (1.08,1.10)	1.10 (1.09,1.11)	1.02 (1.01,1.03)	0.97 (0.96,0.98)	1.00 (0.98,1.02)	1.02 (0.99,1.04)
Unknown/missing	1.24 (1.23,1.25)	1.24 (1.22,1.26)	1.20 (1.18,1.21)	1.20 (1.18,1.21)	1.13 (1.11,1.15)	1.18 (1.14,1.22)
Current smoker vs. former or never	0.90 (0.90,0.91)	0.97 (0.95,0.98)	0.97 (0.96,0.97)	1.01 (1.00,1.03)	1.04 (1.02,1.05)	1.01 (0.99,1.04)
Homeless or history of homelessness	0.78 (0.76,0.79)	0.89 (0.87,0.91)	0.87 (0.85,0.89)	0.99 (0.96,1.01)	0.94 (0.90,0.98)	1.00 (0.96,1.05)
Clinical/Laboratory Measurements						
Body mass index (kg/m^2), per 5 kg/m^2	0.99 (0.99,1.00)	0.99 (0.98,0.99)	1.03 (1.03,1.03)	1.05 (1.04,1.05)	1.03 (1.02,1.03)	1.02 (1.01,1.02)
SBP, per 10 mm Hg	0.94 (0.93,0.94)	0.87 (0.87,0.87)	0.93 (0.93,0.93)	0.87 (0.87,0.88)	0.96 (0.95,0.96)	0.92 (0.91,0.92)
DBP, per 10 mm Hg	0.85 (0.85,0.85)	0.77 (0.77,0.77)	0.88 (0.88,0.88)	0.82 (0.81,0.82)	0.95 (0.94,0.95)	0.96 (0.95,0.97)
Herat rate, per 10 bpm	0.88 (0.88,0.88)	0.91 (0.91,0.92)	0.90 (0.89,0.90)	0.96 (0.95,0.96)	0.91 (0.90,0.91)	0.95 (0.94,0.96)
Total cholesterol (mg/dL), per 10 mg/dL	0.97 (0.97,0.97)	0.94 (0.94,0.94)	0.97 (0.97,0.97)	0.96 (0.95,0.96)	0.99 (0.99,1.00)	0.99 (0.98,1.00)
High-density lipoprotein cholesterol (mg/dL), per 10 mg/dL	0.92 (0.92,0.93)	0.91 (0.90,0.91)	0.92 (0.91,0.92)	0.89 (0.89,0.90)	0.96 (0.95,0.97)	0.96 (0.94,0.97)

© 2025 Derington CG et al. *JAMA Network Open*.

Low-density lipoprotein cholesterol (mg/dL), per 10 mg/dL	0.96 (0.95,0.96)	0.93 (0.93,0.93)	0.96 (0.96,0.96)	0.95 (0.95,0.95)	0.97 (0.97,0.98)	0.98 (0.97,0.99)
Triglycerides (mg/dL), per 10 mg/dL	1.00 (1.00,1.00)	1.00 (1.00,1.00)	1.01 (1.01,1.01)	1.01 (1.01,1.01)	1.01 (1.00,1.01)	1.00 (1.00,1.01)
Hemoglobin A1c (%), per 0.5%	0.99 (0.99,0.99)	1.02 (1.02,1.02)	0.99 (0.99,0.99)	1.02 (1.01,1.02)	1.00 (1.00,1.00)	1.00 (1.00,1.01)
Estimated glomerular filtration rate (mL/min/1.73m^2), per 10 mL/min/1.73m^2	0.93 (0.93,0.93)	0.90 (0.90,0.91)	0.95 (0.95,0.95)	0.95 (0.95,0.95)	0.95 (0.95,0.95)	0.95 (0.95,0.96)
Medical Conditions,						
Alcohol abuse	0.90 (0.89,0.91)	0.84 (0.82,0.87)	1.00 (0.99,1.02)	0.97 (0.95,1.00)	1.13 (1.09,1.18)	1.14 (1.08,1.21)
Chronic kidney disease	1.09 (1.07,1.11)	1.22 (1.19,1.25)	1.04 (1.02,1.06)	1.04 (1.02,1.07)	1.01 (0.97,1.04)	0.94 (0.90,0.98)
Diabetes	0.69 (0.68,0.69)	1.01 (0.99,1.02)	0.68 (0.67,0.69)	0.92 (0.91,0.94)	0.66 (0.65,0.67)	0.77 (0.75,0.79)
Depression	0.77 (0.77,0.78)	0.84 (0.82,0.85)	0.85 (0.84,0.85)	0.96 (0.94,0.97)	0.90 (0.88,0.92)	0.93 (0.90,0.97)
Drug/substance use	0.74 (0.72,0.75)	0.83 (0.80,0.85)	0.85 (0.83,0.87)	0.91 (0.89,0.94)	0.86 (0.81,0.92)	0.90 (0.85,0.96)
End-stage kidney disease or dialysis	2.01 (1.52,2.67)	2.67 (2.41,2.95)	2.11 (1.57,2.83)	2.52 (2.26,2.81)	1.41 (0.77,2.59)	1.93 (1.55,2.39)
Frail based on the VA Frailty index†	1.37 (1.35,1.40)	1.69 (1.66,1.72)	1.24 (1.22,1.27)	1.37 (1.34,1.39)	1.26 (1.22,1.31)	1.26 (1.22,1.30)
Obstructive sleep apnea	0.71 (0.69,0.73)	0.76 (0.74,0.77)	0.77 (0.76,0.79)	0.84 (0.82,0.86)	0.87 (0.84,0.91)	0.87 (0.84,0.91)
Peripheral artery disease	1.18 (1.15,1.20)	1.50 (1.43,1.57)	1.12 (1.10,1.15)	1.27 (1.21,1.33)	1.12 (1.07,1.17)	1.13 (1.05,1.22)
Stroke	1.38 (1.36,1.39)	1.57 (1.53,1.60)	1.27 (1.26,1.29)	1.33 (1.31,1.36)	1.19 (1.16,1.22)	1.08 (1.04,1.12)
Medication Use						
Aspirin	1.63 (1.62,1.64)	2.13 (2.11,2.16)	1.59 (1.58,1.60)	1.94 (1.92,1.96)	1.45 (1.43,1.48)	1.57 (1.54,1.61)
Statin	1.50 (1.50,1.51)	1.78 (1.76,1.79)	1.44 (1.44,1.45)	1.61 (1.60,1.63)	1.31 (1.29,1.32)	1.35 (1.33,1.38)
Healthcare Utilization in Prior Year						
Primary care visits, per 1 visit	0.96 (0.96,0.96)	0.99 (0.99,1.00)	0.97 (0.96,0.97)	0.99 (0.99,1.00)	0.98 (0.98,0.99)	1.00 (0.99,1.00)
Hospitalizations, per 1 hospitalization	1.09 (1.09,1.10)	1.07 (1.05,1.08)	1.13 (1.12,1.13)	1.08 (1.07,1.09)	1.18 (1.17,1.20)	1.06 (1.02,1.09)
Emergency department visits, per 1 visit	0.93 (0.93,0.94)	0.95 (0.94,0.95)	0.96 (0.96,0.97)	0.98 (0.97,0.98)	0.98 (0.97,0.99)	0.99 (0.97,1.01)
Facility characteristics						
Veterans Integrated Service Network Region#						
Northeast	1 (Ref)	1 (Ref)	1 (Ref)	1 (Ref)	1 (Ref)	1 (Ref)
Southeast	0.82 (0.82,0.83)	0.83 (0.82,0.84)	0.85 (0.84,0.85)	0.89 (0.88,0.90)	0.90 (0.89,0.91)	0.93 (0.92,0.95)
Continental	0.92 (0.91,0.92)	0.89 (0.88,0.90)	0.94 (0.93,0.94)	0.93 (0.92,0.94)	0.94 (0.93,0.96)	0.95 (0.93,0.98)
Pacific	0.89 (0.88,0.89)	0.83 (0.82,0.84)	0.91 (0.90,0.91)	0.85 (0.84,0.86)	0.93 (0.91,0.94)	0.90 (0.88,0.93)
Academic vs. non-academic setting	0.99 (0.98,0.99)	0.94 (0.93,0.95)	0.99 (0.99,1.00)	0.95 (0.94,0.96)	1.02 (1.01,1.03)	0.96 (0.95,0.98)
Urban vs. rural setting	1.06 (1.05,1.07)	1.09 (1.07,1.11)	1.06 (1.05,1.07)	1.06 (1.04,1.08)	0.99 (0.96,1.02)	1.01 (0.97,1.05)

© 2025 Derington CG et al. *JAMA Network Open*.

*Includes Asian American, American Indian or Alaskan Native, and Native Hawaiian or Other Pacific Islander.

ψAccording to the US Bureau of Economic Analysis, matched to ZIP or FIPS code (https://apps.bea.gov/regional/histdata/index.cfm)

† Ratio of the sum of the number of health deficits relative to 31 health factors evaluated, ranging from 0 to 1. 0 indicates no frailty, while non-zero numbers indicate some degree of frailty. Non-frail was defined as a calculated frailty index ≤0.21, and frail was defined as a calculated frailty index >0.21.

Categorized according to the VA regional offices map (https://www.benefits.va.gov/benefits/offices.asp). The Northeast region was comprised of VISNs 1, 2, 4, 5, 10, and 12. The Southeast region was comprised of VISNs 6, 7, 8, 9, and 16. The Continental region consisted of VISNs 15, 17, 18, 19, and 23. Finally, the Pacific region was comprised of VISNs 20, 21, and 22

© 2025 Derington CG et al. *JAMA Network Open*.
