# Supplementary material for: First-Line β-Blocker Use for Hypertension in the Veterans Health Administration
Source: JAMA Netw Open. 2025 Aug 27;8(8):e2529026. doi: 10.1001/jamanetworkopen.2025.29026 (PMC12391979; doi:10.1001/jamanetworkopen.2025.29026)
Supplement: Supplement 2. — Data Sharing Statement [file jamanetwopen-e2529026-s002.pdf]

Data Sharing Statement

Derington. First-Line β-Blocker Use in the Veterans Health Administration. *JAMA Netw Open*. Published August 27, 2025. doi:10.1001/jamanetworkopen.2025.29026

Data
Data available: No
